# Diagnostic delay of sarcoidosis: an integrated systematic review

**DOI:** 10.1186/s13023-024-03152-7

**Published:** 2024-04-11

**Authors:** Tergel Namsrai, Christine Phillips, Anne Parkinson, Dianne Gregory, Elaine Kelly, Matthew Cook, Jane Desborough

**Affiliations:** 1 National Centre for Epidemiology and Population Health, The Australian National University, 63, Eggleston Road, Acton ACT, Canberra, 2601 Australia; 2 Sarcoidosis Lyme Australia, Camden, Australia; 3 John Curtin School of Medical Research, The Australian National University, Canberra, Australia; 4 School of Medicine and Psychology, The Australian National University, Canberra, Australia

**Keywords:** Sarcoidosis, Diagnostic delay, Misdiagnosis, Systematic review, Meta-analysis, Meta-aggregation, Rare disease

## Abstract

**Background:**

Sarcoidosis is a chronic inflammatory granulomatous disease of unknown cause. Delays in diagnosis can result in disease progression and poorer outcomes for patients. Our aim was to review the current literature to determine the overall diagnostic delay of sarcoidosis, factors associated with diagnostic delay, and the experiences of people with sarcoidosis of diagnostic delay.

**Methods:**

Three databases (PubMed/Medline, Scopus, and ProQuest) and grey literature sources were searched. Random effects inverse variance meta-analysis was used to pool mean diagnostic delay in all types of sarcoidosis subgroup analysis. Diagnostic delay was defined as the time from reported onset of symptoms to diagnosis of sarcoidosis.

**Results:**

We identified 374 titles, of which 29 studies were included in the review, with an overall sample of 1531 (694 females, 837 males). The overall mean diagnostic delay in all types of sarcoidosis was 7.93 months (95% CI 1.21 to 14.64 months). Meta-aggregation of factors related to diagnostic delay in the included studies identified three categories: (1) the complex and rare features of sarcoidosis, (2) healthcare factors and (3) patient-centred factors. Meta-aggregation of outcomes reported in case studies revealed that the three most frequent outcomes associated with diagnostic delay were: (1) incorrect diagnosis, (2) incorrect treatment and (3) development of complications/disease progression. There was no significant difference in diagnostic delay between countries with gatekeeper health systems (where consumers are referred from a primary care clinician to specialist care) and countries with non-gatekeeper systems. No qualitative studies examining people’s experiences of diagnostic delay were identified.

**Conclusion:**

The mean diagnostic delay for sarcoidosis is almost 8 months, which has objective consequences for patient management. On the other hand, there is a paucity of evidence about the experience of diagnostic delay in sarcoidosis and factors related to this. Gaining an understanding of people’s experiences while seeking a diagnosis of sarcoidosis is vital to gain insight into factors that may contribute to delays, and subsequently inform strategies, tools and training activities aimed at increasing clinician and public awareness about this rare condition.

**Trial registration:**

PROSPERO Registration number: CRD42022307236.

**Supplementary Information:**

The online version contains supplementary material available at 10.1186/s13023-024-03152-7.

## Introduction

Sarcoidosis is a multisystem granulomatous inflammatory disease of unknown cause, which can affect any organ, but primarily affects the lungs. Sarcoidosis can present as acute or chronic disease - acute sarcoidosis, with joint pain, erythema nodosum and hilar adenopathy, that resolves spontaneously; or chronic sarcoidosis with insidious onset and slow progression that continues to invade multiple systems. In studies using national patient registers the incidence appears to be highest in northern Europe at 11.5 per 100,000 per year in Sweden [1] and 11.3–14.8 per 100,000 per year in Denmark [2], There are significant intra-country differences attributable to ethnicity in the USA where African Americans have significantly higher rates of disease [1, 2], earlier peak age of onset [3] and higher mortality [4]. The patterns of organ involvement [5–7] and gender distribution [3, 4, 8] vary between countries and within countries.

The reported delay of diagnosis in sarcoidosis ranges from 6 months [2] to 24 months [9]. Its complex clinical features, acute or chronic presentation, spontaneous or treatment-induced remission in some cases, and the absence of a single simple diagnostic test all contribute to challenges in timely diagnosis. In many cases, diagnosis hinges on tissue diagnosis and therefore a firm diagnosis might be delayed even when the diagnosis is suspected. Some of the most consequential extrapulmonary manifestations of sarcoidosis – neurological, ophthalmic, and cardiac – are among the most difficult to diagnose [8–11]. Spontaneous remission occurs frequently in sarcoidosis [12]; some studies report remission in half of the cases [13]. Diagnostic delay can occur with both acute and chronic presentations of sarcoidosis, but particularly for chronic presentations marked by slow progression and complex features, mimicking other diseases. Failure to initiate treatment for progressive pulmonary sarcoidosis [14] and many extrapulmonary manifestations of sarcoidosis can result in permanent organ damage [11, 15, 16]. Since the pathogenesis of sarcoidosis remains unknown, it is a diagnosis of exclusion. The differential diagnosis includes other causes of granulomas, which encompass infections, including mycobacteria, fungi and bacteria, occupational exposures such as beryllium and silica, sterile granulomatous inflammation, and lymphoma.

There is a paucity of research examining the diagnostic delay of sarcoidosis, including factors associated with diagnostic delay and people’s experiences from the time of symptom onset to diagnosis. Our aim was to systematically review the current evidence regarding the diagnostic delay of sarcoidosis and people’s experiences of this. This evidence may help to inform the development of strategies to enhance awareness of rare manifestations of sarcoidosis, enabling timely intervention when warranted for chronic and progressive sarcoidosis.

## Methods

This systematic review was performed and reported in accordance with the Preferred Reporting Items for Systematic Reviews and Meta-Analyses (PRISMA) [17] and the Cochrane Handbook for Systematic Reviews [6]. It is registered with PROSPERO, an International prospective register of systematic reviews (registration number: CRD42022289830).

### Literature search, study selection, and data extraction

A systematic electronic search of the literature was conducted using PubMed/Medline, Scopus, and ProQuest databases up to the 25th of May 2022, with no limitations. The search string was pre-developed and peer-reviewed using the PRESS checklist [18]. The final search string included “sarcoidosis” AND “delay in diagnosis” OR “diagnostic delay” OR “misdiagnosis” OR “time to diagnosis” OR “incorrect diagnosis” OR “missed diagnosis” OR “delayed diagnosis” without restrictions on study type, date, and language. A detailed search string and strategy are available in the published protocol [19]. Grey literature sources were searched up to the 25th of May 2022 in Open Access Theses and Dissertations (https://oatd.org/), ProQuest thesis and dissertations, and the National Library of Australia. Manual reference searches were conducted on all review articles identified in the literature search.

There was no restriction on publication dates. All studies, both qualitative and quantitative, examining diagnostic delay, incorrect diagnosis, missed diagnosis or slow diagnosis of sarcoidosis in all age groups were included, except for review articles. Studies in languages other than English, German and Indonesian were excluded. Final search results were imported into a systematic review management software (Covidence) to facilitate reviewer collaboration [20].

Two authors conducted an independent screening of titles and abstracts followed by a full-text screening of articles using pre-developed PICOS eligibility criteria outlined in Table 1. Articles that did not meet the eligibility criteria were excluded. Discrepancies were resolved in discussion with a third reviewer and through reaching a consensus. Included studies were quality appraised using the Mixed Methods Appraisal Tool (MMAT) [21]. A pre-developed and pre-piloted data extraction tool was used, and following further discussion after piloting, data describing the initial specialist and the presence/absence of gatekeeper health systems were also extracted.

**Table 1:** PICOS eligibility criteria

PICOS	Inclusion criteria	Exclusion criteria
Population	Studies examining people with sarcoidosis of all ages	-
Intervention/Exposure	Studies examining delayed, incorrect diagnosis, missed diagnosis or slow diagnosis of sarcoidosis	-
Comparison	Not applicable	-
Outcome	Primary outcome: diagnostic delay. Secondary outcomes: i) causes and consequences of diagnostic delay ii) people with sarcoidosis’ experiences of diagnostic delay	-
Study design	All study designs	Review articles
Language	English, German, Indonesian	
Setting	No restriction	-
Timing	No restriction	-

### Data analysis

#### General data preparation

Diagnostic delay was defined in accordance with the included studies - from reported onset of symptoms to a diagnosis of sarcoidosis. In studies where mean diagnostic delay was presented in years or days, we converted it to months. For studies that did not report a standard deviation (SD) of mean diagnostic delay, we imputed the SD using the method recommended by Cochrane, which calculates SD using an upper limit, lower limit, and confidence interval [6]. In studies where the confidence interval was not reported, we calculated SD using the method improved by Wan and colleagues, incorporating the sample size or population [22].

Categorisation of studies was based on the location or organ involvement of sarcoidosis - pulmonary, extrapulmonary, and systemic. Where sarcoidosis involved only the lungs (defined as changes in hila, mediastina, and the lungs) the location was categorised as pulmonary; where sarcoidosis involved two or more organs the location was categorised as systemic. If only one organ other than the lungs was involved, the location was categorised as extrapulmonary. Health systems were categorised as either gatekeeper (where primary care physicians authorise access to specialist physicians) or non-gatekeeper health systems, based on the dominant health system in the country where the study was conducted. A country was classified as having a gatekeeper system if the system of health financing uniformly used primary care gatekeepers, without the option of self-funding to see specialists, or models of health funding that supported open access to specialists. In countries with diverse health insurance models which may include open access and gatekeepers, such as the USA, an assessment was made for each publication by two authors. Where we could not determine the gatekeeper system used by participants the paper was excluded. We calculated the missing mean age of the study sample when complete data of the study participants was available.

#### Analysis of diagnostic delay in sarcoidosis

We used an inverse variance weighted random effects model (Der-Simonian-Laird method) to pool mean diagnostic delay [6]. Sensitivity analyses between studies with estimated SDs and original SDs were conducted. Additionally, we conducted subgroup analyses based on healthcare system type and publication year to investigate possible group differences in diagnostic delay in sarcoidosis. We analysed quantitative data through a meta-synthesis. The alpha level was set at 0.05, and the heterogeneity of meta-analysis estimates was presented using the I^2^ statistic. Funnel plots were used to assess the risk of publication bias.

We descriptively analysed and presented a narrative synthesis of the quantitative data from case reports that could not be pooled. Gender difference in diagnostic delay was calculated in case reports where data on sex and delay in diagnosis (months) was available. The distribution of delay in diagnosis in case reports was examined by density plot and Shapiro test (*p* <.05), indicating non-normal distribution; thus, the Mann-Whitney-Wilcoxon test was used to analyse the group differences of delay in diagnosis by sex. All statistical analyses were performed using R version 4.6.2 [23] and the ‘meta’ package.

#### Analysis of symptoms, factors, outcomes and experiences associated with diagnostic delay

To investigate the factors associated with diagnostic delay, data on symptoms that changed the diagnosis, and factors related to and outcomes of diagnostic delay were extracted and synthesised using meta-aggregation, for which meanings from qualitative data are identified and aggregated into categories that can be synthesised and analysed [24]. The broader categorisation of the aggregated data was decided through peer discussion and referral back to the original papers when needed. Additionally, factors linked to pulmonary, extrapulmonary and systemic sarcoidosis were grouped and further analysed.

To our knowledge, none of the included studies reported data on experiences of diagnostic delay in sarcoidosis.

## Results

**Fig. 1:**
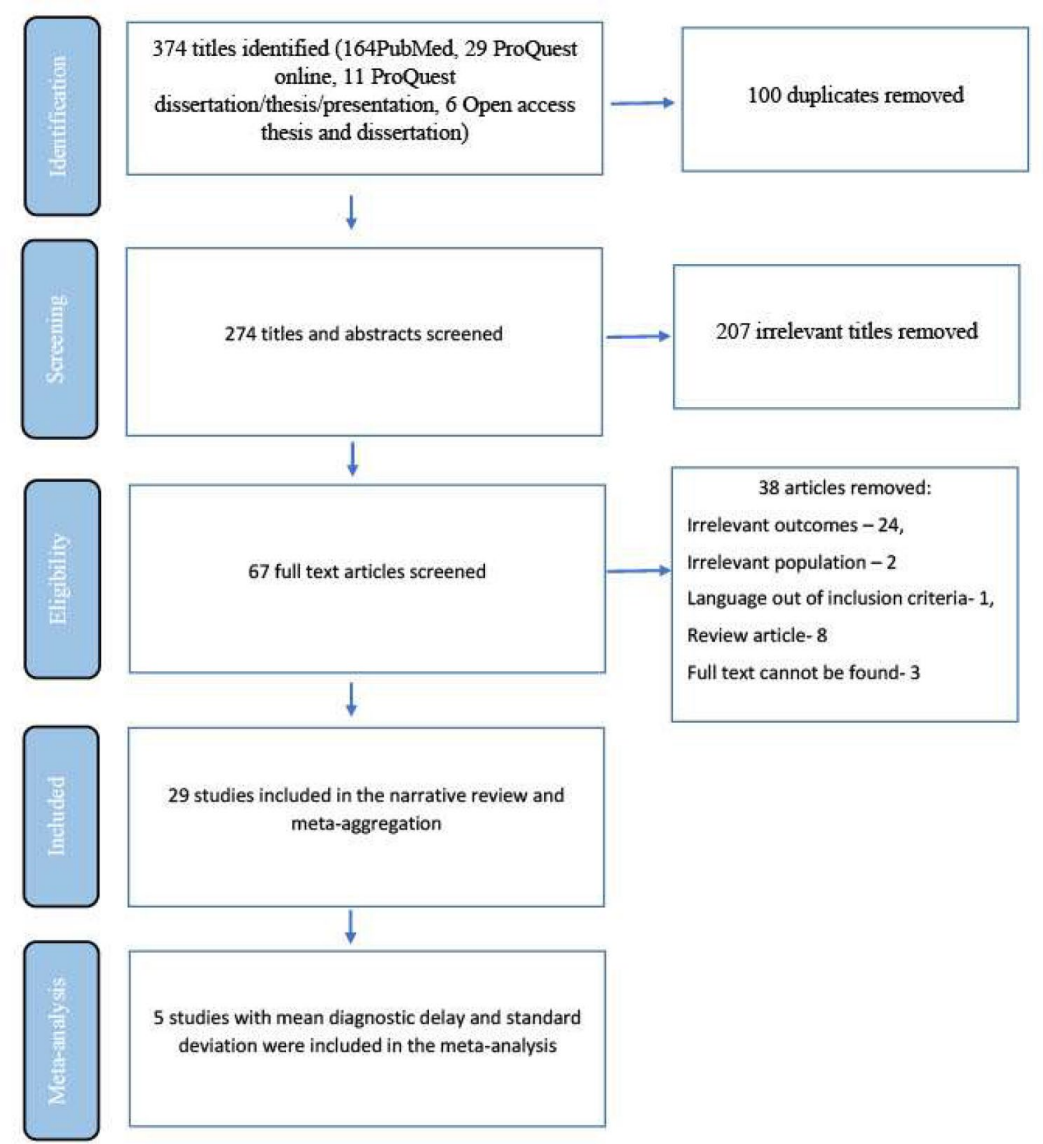
Selection flow chart of studies included in the systematic review

Out of 374 titles identified, we removed 100 duplicates, and screened 274 titles and abstracts. Of those, 67 articles were reviewed at full text and 29 studies were included in the review as shown in Fig. 1.

### Description of included studies

Included studies are summarised in Table 2 and a full data extraction table is presented in Supplementary Table 1. The 29 included studies comprised 24 non-comparative descriptive studies (including 15 case reports [25–39], five case series [40–44], two surveys [45, 46], and two descriptive cross-sectional studies [2, 47]), and five comparative studies (all analytical cross-sectional studies) [9, 48–51]. Twenty-eight of the included studies used non-patient-reported data including clinical reports and retrospective patient registry data, while one used patient-reported data [45]. In total, there were eleven studies from Europe [2, 9, 25, 26, 33, 35, 38, 42, 45, 47, 48], nine from the United States or Canada [27, 29, 30, 32, 34, 37, 43, 44, 49], three from West Asia [41, 46, 50], four from East Asia [28, 31, 39, 40], one from sub-Saharan Africa [36], and one from South America [51]. Various organ involvement of sarcoidosis was reported, including eyes [9], nasal passages [42], kidney [26, 27], skin [28, 34], heart [40, 48], nervous system [30, 38, 44], lungs [35–37, 43], skeletal muscle [33], subcutaneous tissue [39], and systemic or mixed [2, 25, 29, 31, 32, 41, 45, 46, 49–51]. Based on the manual categorisation, thirteen studies examined extrapulmonary sarcoidosis [26–30, 32–34, 39, 40, 44, 47, 48], five examined systemic sarcoidosis [9, 25, 31, 38, 42], and four examined pulmonary sarcoidosis [35–37, 43]. In seven studies it was not possible to differentiate between pulmonary and non-pulmonary sarcoidosis [2, 41, 45, 46, 49–51]. Of the 29 included studies, 18 were from countries with non-gatekeeper health systems (2, 25, 27–33, 3537, 39, 40, 43, 44, 47, 49) and 11 were from countries with gatekeeper health systems [9, 26, 34, 38, 41, 42, 45, 46, 48, 50, 51]. Twelve studies reported data on ethnicity or race [2, 25, 29–33, 36, 42, 43, 49, 51].

**Table 2:** Descriptive table of selected studies categorised according to study designs based on MMAT algorithm

Author	Country	Sample size (n)	Patient reported data (Yes/No)	Gender (n; Male/ Female)	Mean age (year)	Mean delay (months)	Mean delay SD^a^ (months)
	**1. Non-comparative descriptive study including case reports, case series, survey and descriptive cross-sectional studies**						
	a. Case reports						
Darugar et al., 2011	France	1	No	1/0	26	0.5	NR
Froehner et al., 2016	Germany	1	No	1/0	60	6	NR
Ghafoor et al., 2014	USA	1	No	1/0	69	44	NR
Ghorpade et al., 1996	India	1	No	0/1	50	2	NR
Ho et al., 2019	USA	1	No	1/0	55	2	NR
Jaster et al., 1997	USA	1	No	1/0	41	18	NR
Lee et al., 2010	South Korea	1	No	0/1	32	59	NR
Mehta et al., 2022	USA	1	No	1/0	49	8.5	NR
Meyer et al., 2017	Switzerland	1	No	1/0	52	48	NR
Noiles et al., 2013	Canada	1	No	0/1	62	Several years	NR
Papaetis et al., 2008	Greece	1	No	0/1	67	96	NR
Plit 1983	South Africa	1	No	0/1	27	18	NR
Thomas et al., 2021	USA	1	No	0/1	48	0.5	NR
van Rooijen et al., 2011	Netherlands	1	No	0/1	30	1.4	NR
Viswanath et al., 2019	India	1	No	0/1	50	0.25	NR
	b. Case series						
Al-Mayouf 2006	Saudi Arabia	8	No	2/6	9.3	6	4.03
Fergie et al., 1999	UK	8	No	2/6	44	5	5.92
Guleria et al., 2006	India	3	No	2/1	40.7	Case 1- 1.5 Case 2–18 Case 3–6 Case 4- NR Mean – 8.5	NR
Judson et al., 2007	USA	2	No	1/1	37	Case 1–15 Case 2–72 Mean – 43.5	NR
Scott et al., 2010	USA	8	No	0/8	NR	NR	NR
	c. Survey						
Kirsten et al., 1995	Germany	651	Yes	243/408	NR	25	NR
Okumus et al., 2011	Turkey	293	No	95/198	44	NR	NR
	d. Descriptive cross-sectional study						
Leclerc et al., 2003	France	28	No	17/11	NR	6.25	3.72
Send et al., 2019	Germany	13	No	6/7	48.8	8.61	6.4
	**2. Comparative studies including analytical cross-sectional study**						
	a. Analytical cross-sectional study						
Bolletta et al., 2020	Italy	67	No	29/38	55	23	35
Hoogendoorn et al., 2020	Netherlands	15	No	9/6	50.7	NR	NR
Judson et al., 2003^c^	USA	189	No	81/108	NR	NR	NR
Kobak et al., 2020	Turkey	131	No	35/96	NR	NR	NR
Rodrigues et al., 2013^c^	Brazil	100	No	40/60	47.6	NR	NR

^a^SD- Standard deviation

^b^NR- Not reported - data that has not been reported in the original study was described as NR

^c^Studies that used statistical method to compare, measure or explore the link between diagnostic delay and possible factors in the one or more groups

In total, a population size of 1531 participants (694 females; 837 males) was included in the review. The mean age was 47.91 years (SD = 5.47), excluding case reports (see below). Overall, participant ages ranged from 9.3 years to 69 years (including case reports).

### Results of the quality appraisal

Consensus on the quality appraisal of the included studies is shown in Supplementary Table 2. After the double-quality appraisal, a consensus was reached by two authors regarding an overall low risk of bias for all studies; therefore, no study was excluded.

### Case studies

Twenty case studies comprising 15 case reports (8 females; 7 males) [25–39] and five case series [40–44], with 29 participants (22 females, 7 males), were included. The mean age of participants in case report studies was 47.87 years (SD = 14.06 years), with individual age ranging between 26 years [25] and 69 years [27]. In the case series, mean age of individuals ranged from 9.3 years [41] to 44 years [42].

Of the 20 included case studies, 11 examined extrapulmonary sarcoidosis [26–30, 32–34, 39, 40, 44], and four each focused on pulmonary [35–37, 43] and systemic sarcoidosis [25, 31, 38, 42]. In the one remaining case study, it was not possible to determine the extent of organ involvement [41].

In the 15 included case reports, individual diagnostic delay ranged from 0.25 months (0.02 years) [39] to 96 months (8 years) [35] and the mean diagnostic delay was 21.73 months. In the five case series, the mean diagnostic delay ranged from 5 months [42] to 43.5 months [43].

There was no significant gender difference in delay in diagnosis in case reports (*n* = 15, Mann-Whitney-Wilcoxon test: w = 21.5, *p* =.749).

### Pooled diagnostic delay in sarcoidosis

**Fig. 2:**
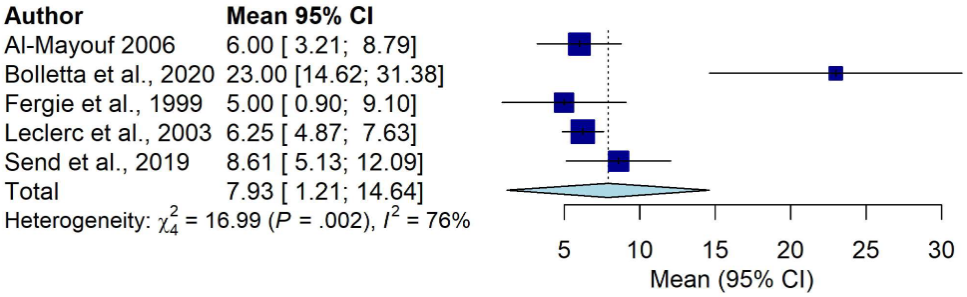
Pooled mean diagnostic delay in sarcoidosis

The results of the pooled mean diagnostic delay of the five studies [2, 9, 23, 41, 42] with an overall sample size of 124 are presented in Fig. 2. Individual study sample size of these studies ranged from 8 [41, 42] to 67 [9], while the mean diagnostic delay ranged from 5 months [42] to 23 months [9]. The pooled diagnostic delay was 7.93 months (95% CI 1.21 to 14.64 months) **(**Fig. 2**)**. A funnel plot of the pooled diagnostic delay is presented in Supplementary Fig. 1. We conducted a sensitivity analysis on SD estimated studies and SD not estimated studies and found no significant difference (between groups difference = 1.06 months, *P* =.30) in mean diagnostic delay between the two groups as shown in Supplementary Fig. 2.

We could not conduct a subgroup analysis between pulmonary, extrapulmonary and systemic sarcoidosis due to the small number of studies with complete data (mean delay, total number of participants and SD of mean delay) in each group. However, in the included studies, systemic sarcoidosis had the longest mean diagnostic delay at 23.0 months [9] compared with extrapulmonary sarcoidosis, which had the shortest mean diagnostic delay of 5.0 months [42].

A subgroup analysis comparing studies (*n* = 5) with different healthcare systems is presented in Supplementary Fig. 3. There was no significant difference in mean diagnostic delay in countries with gatekeeper healthcare systems when compared with those with non-gatekeeper systems (between groups difference = 0.34 months, *P*_=_.56).

We conducted an additional subgroup analysis examining publication year of studies, which showed a significant inter-study difference in diagnostic delay in studies conducted (between groups difference = 16.99 months, *P* =.002) (see Supplementary Fig. 4). Further analysis examining publication year of the studies (e.g., before 2000 and after 2000) was not feasible due to the small number of studies.

### Initial symptoms

Twenty-one studies comprising 15 case reports [25–39], three case series [40, 42, 43], two cross-sectional studies [47, 49] and one survey [46] reported initial symptoms. Initial symptoms included weight loss [29, 36, 37, 41, 43], fatigue or generalised weakness [29, 37, 40, 43], dyspnoea [36, 40, 43], muscle pain/muscle cramps/general body pain [32, 37, 40], headache [38], palpitations [40], nasal obstruction [42] and a subcutaneous mass [39] (refer to Supplementary Table 3). When aggregated, these symptoms could be categorised as: (1) general symptoms (fever, fatigue, weight loss), (2) organ-specific extrapulmonary symptoms (neurological- nausea, headache, vomiting; cardiac- palpitations; skin - rash, ulcers), and (3) pulmonary symptoms (cough, dyspnoea). Of the initial symptoms, 31.25% (25/80) were general; 55% (44/80) were organ specific and related to extrapulmonary symptoms, while 13.75% (11/80) were pulmonary (see Supplementary Table 3).

### Initial specialist and treatment/diagnostic centre

Five of the included studies reported the cadre of specialist first consulted, one study each reporting general practitioner [29], emergency specialist [31], gynaecologist [38], oncologist [39], and neurologist [32] as the first specialist consulted. Twenty-one studies reported visits to treatment or diagnostic centres including secondary or tertiary hospitals, research centres and university hospitals [2, 9, 25–27, 31–33, 35, 37–42, 44, 47–51]. Nineteen of these 21 studies reported treatment or diagnosis at multidisciplinary centres [2, 9, 25–27, 31–33, 35, 37, 38, 41, 42, 44, 47, 48, 50, 51], and one study each at an institute of oncology [39] and a research centre [49].

### Symptoms that changed the diagnostic approach

Twelve case studies, containing a total of 13 cases/participants, reported 24 symptoms that changed the diagnostic approach [27, 31–38, 40, 43]. These symptoms ranged from no response to treatment [31, 36, 43], persistent or increasing shortness of breath/dyspnoea [35, 40, 43], persistent cough [35, 37] to worsening hypertension [27], renal function decline and hypercalcemia [27] (Supplementary Table 4). None of the cross-sectional studies and surveys reported symptoms that changed the diagnostic approach. When aggregated, symptoms that changed the diagnostic approach were categorised into: (1) persistent symptoms (7/24, 29.2%) [33, 35, 40, 43], (2) new symptoms or signs (7/24, 29.2%) [31, 32, 38], (3) worsening of symptoms (6/24, 25%) [27, 34, 40] and (4) no response to treatment (4/24, 16.6%) [31, 36, 43], as shown in Supplementary Tables 4 and Supplementary Fig. 5.

### Factors related to diagnostic delay

Fifteen case reports [25–39], three case series [40, 42, 44], two analytical cross-sectional studies [9, 48], one survey [45] and one descriptive cross-sectional study [47] reported factors that might influence diagnostic delay in sarcoidosis (see Supplementary Table 5). Two analytical cross-sectional studies examined association between several factors and diagnostic delay [49, 51]. In one study, the presence of pulmonary symptoms was associated with a longer time to diagnosis, whereas the presence of skin symptoms was associated with a shorter time to diagnosis [49]. People assessed as being at a higher stage on the Scadding scale (radiological scale to measure lung changes; higher stage correlates to greater structural damage in lungs) had a longer time-to-diagnosis compared to people with lower stage features (stage IV vs. stage II, stage III vs. stage 0 or I on chest radiographs) [49]. One study in Brazil found that misdiagnosis of and treatment for tuberculosis was more likely to be reported among those with a time-to-diagnosis of more than 6 months [51].

**Fig. 3:**
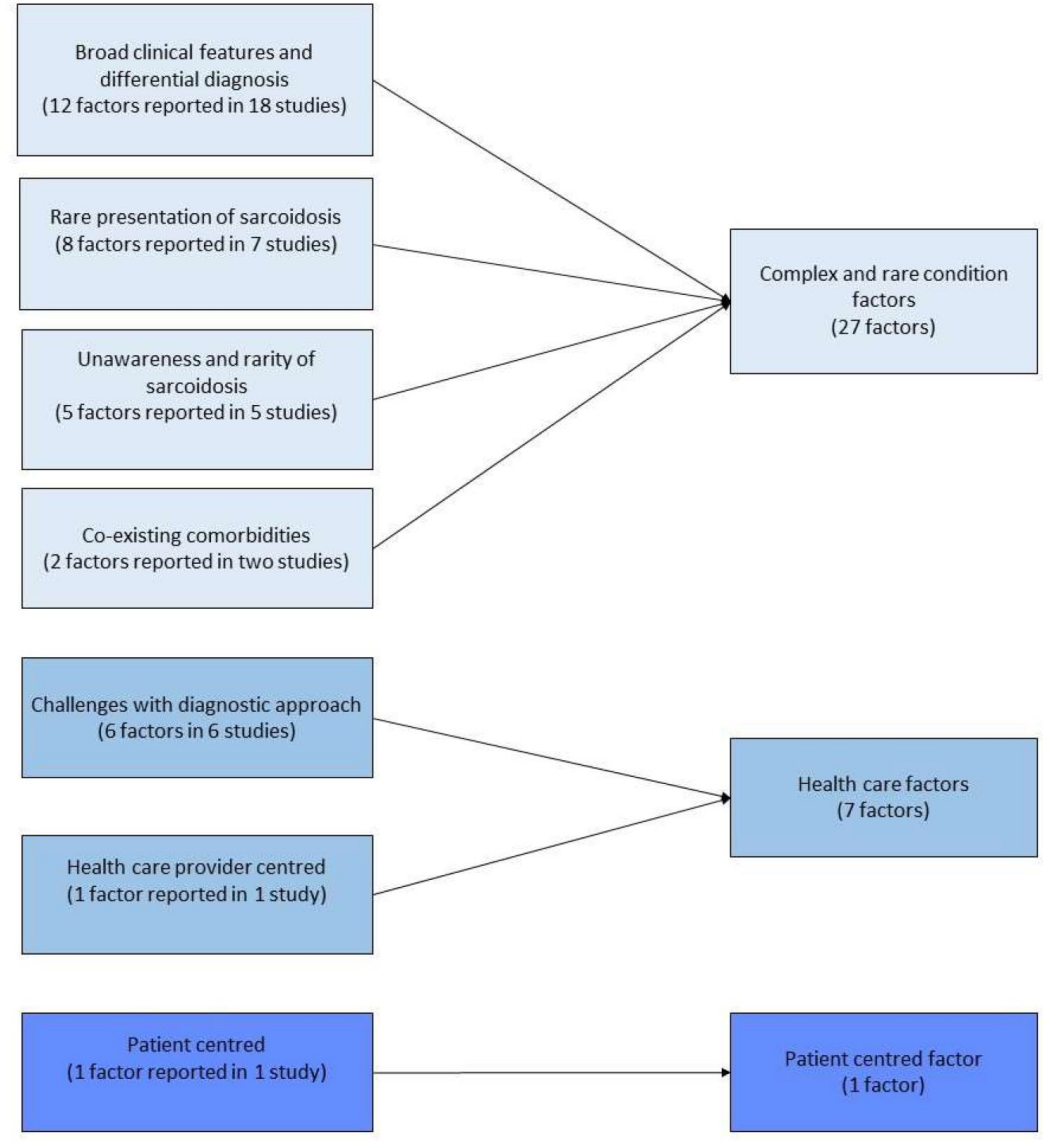
Meta-aggregation results of factors related to diagnostic delay in sarcoidosis

The factors mentioned in the 22 studies were meta-aggregated and the results are shown in Fig. 3. We categorised these factors into: (1) complex and rare features of sarcoidosis (27/35, 77.1%), (2) healthcare factors (7/35, 20%) and (3) patient-centred factors (1/35, 2.9%). Of these, 77.1% (27/35 factors) were related to complex and rare features of sarcoidosis (category 1), including broad clinical features and differential diagnosis [9, 25–28, 31–38, 40, 42, 44, 47, 48], rare presentation [28, 32, 34, 36–38, 42], lack of awareness and rarity of sarcoidosis [29, 30, 32, 33, 40], and coexisting disease or comorbidities [35, 38]. The 20% (7/35 factors) pertaining to healthcare factors (category 2) included exclusion diagnosis [39], lack of standard procedure to distinguish sarcoidosis [47], not using appropriate diagnostic techniques/ relying on chest x-ray [45, 48], challenges with biopsy [9, 40], and challenges with making a definitive diagnosis in sarcoidosis [32]. The remaining 2.9% of factors were patient-centred (category 3), which referred to refusal of biopsy (1/35 factors) [35].

We further analysed these factors by sarcoidosis type (Supplementary Table 6). Twenty-two studies reported types of sarcoidosis; of these, 21 studies [9, 25–40, 42, 44, 47, 48] reported factors related to diagnostic delay. Three studies reported five factors of diagnostic delay in pulmonary sarcoidosis. Of these, 80% were categorised as complex and rare features of sarcoidosis (category 1), which included co-existing disease and comorbidities [35], rare presentations [36, 37] and broad clinical features [37]. The remaining 20% were patient-centred factors, referring to patient’s refusal of a biopsy (category 3) [35]. Thirteen studies reported twenty factors related to diagnostic delay in extrapulmonary sarcoidosis [26–30, 32–34, 39, 40, 44, 47, 48]. Of these, 75% were linked to complex and rare features of sarcoidosis (category 1), including broad clinical features and differential diagnosis [26–28, 40, 44, 47, 48], rare presentation [28, 32, 34] and lack of awareness of sarcoidosis [29, 30, 32, 33, 40]. The remaining 25% were categorised as healthcare related (category 2), which included factors relating to healthcare providers [48] and challenges with diagnostic approach or tools [32, 39, 40, 47]. Nine factors were mentioned to be linked to diagnostic delay in systemic sarcoidosis [9, 25, 31, 38, 42]; eight of these were linked to the complex and rare features of sarcoidosis (category 1); broad clinical features [25, 31, 38, 42], rare presentation [9, 38, 42], and co-existing disease [38]. One factor was linked to healthcare (category 2): challenges with diagnostic approach and tool, described by the authors of the paper as limited number of patients amenable to lymph node biopsy [9].

### Outcomes related to diagnostic delay

Sixteen studies described the outcomes of diagnostic delay, including 11 case reports [26, 27, 31–39], two case series [40, 44], two analytical cross-sectional studies [48, 51], and one survey [45]. The survey and analytical cross-sectional studies did not use statistical methods to examine the relationship between independent variables and diagnostic delay; however, they reported descriptive or comparative results of the outcomes of diagnostic delay. One study described incorrect diagnoses that were provided instead of sarcoidosis, including tuberculosis, lung cancer, rheumatic fever, Hodgkin’s lymphoma, pneumonia, and patients simulating the symptoms [45]. One study reported irreversible deterioration of cardiac function (6/10 cases) and high mortality (5/10 cases) in people with a late diagnosis of sarcoidosis [48], and another study reported poor lung function in people with a late diagnosis [51].

**Fig. 4:**
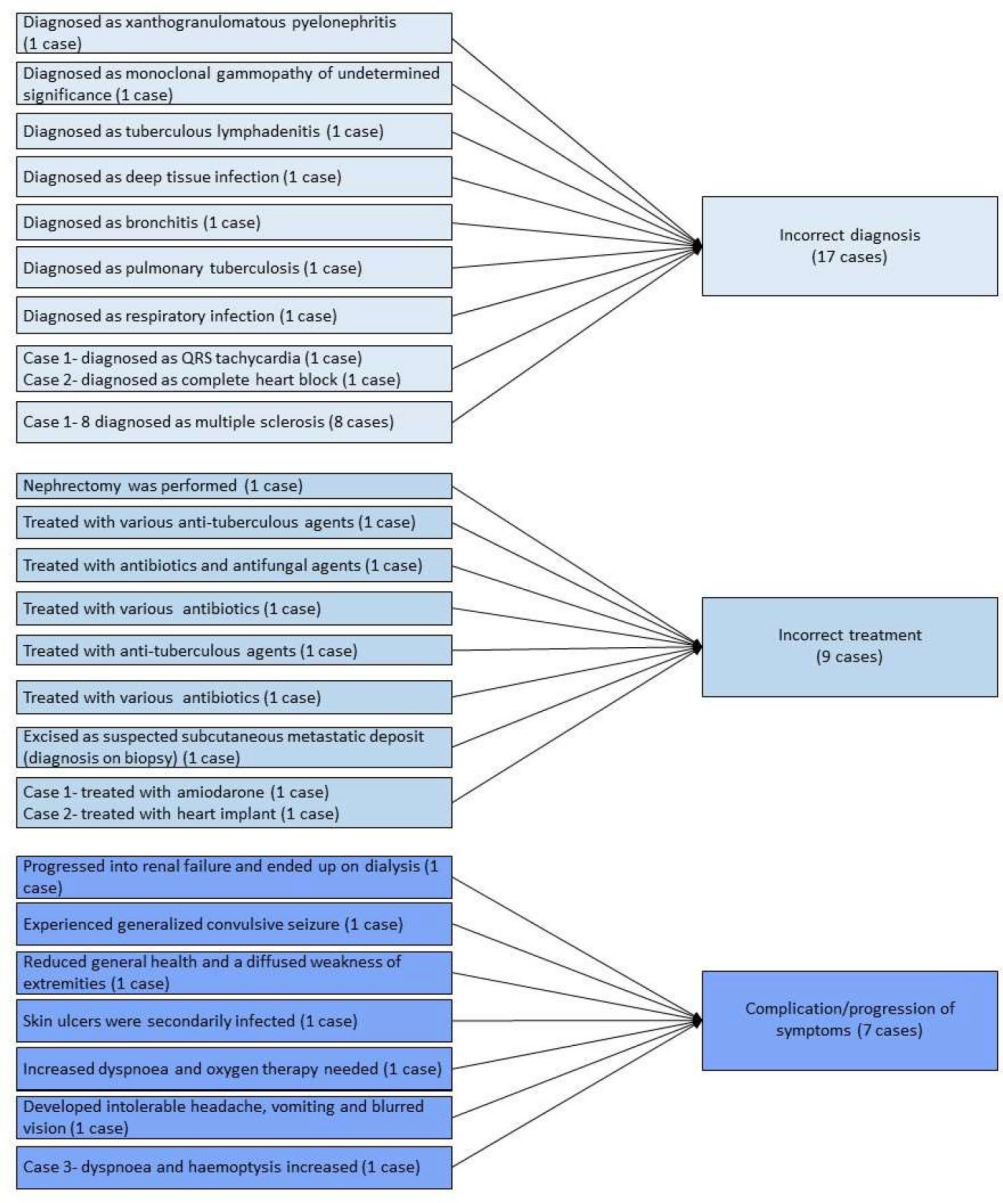
Meta-aggregation of outcomes of diagnostic delay in case studies

While case reports or case studies are not designed to assess the association between two variables, we analysed their data using meta-aggregation as shown in Supplementary Tables 7 and Fig. 4. Thirteen case studies, including 11 case reports [26, 27, 31–39] and two case series [40, 44], described outcomes from 26 cases. We aggregated the outcomes into: (1) incorrect diagnosis, (2) incorrect treatment and (3) complications/progression of the condition. Incorrect diagnosis (category 1) was reported in 38.5% (10/26 cases), including xanthogranulomatous pyelonephritis [26], monoclonal gammopathy of undetermined significance [27], tuberculosis [31, 36], deep tissue infection [34], bronchitis [35], respiratory infection [37], tachycardia and heart block [40], and multiple sclerosis [44]. Incorrect treatment (category 2) was reported in 34.6% (9/10 cases), consisting of nephrectomy [26], anti-tuberculosis agents [31, 36], antibiotics [34, 35, 37] and excision of mass [39]. Complications/progression of symptoms or the condition (category 3) was reported in 26.9% (7/10 cases) of the cases. These included renal failure [27], seizure [32], weakness of the extremities [33], infection [34], dyspnoea and oxygen therapy [35], headache, vomiting and blurred vision [38], dyspnoea and haemoptysis [40].

### People’s experiences related to diagnostic delay

We did not identify any studies, including qualitative, that examined people’s experiences of diagnostic delay in our systematic search.

## Discussion

Using data from the 29 studies included in this review, we were able to present a pooled analysis of diagnostic delay in all types of sarcoidosis to describe factors that are related to and associated with diagnostic delay, and the outcomes for people living with sarcoidosis. Pooled mean diagnostic delay for all types of sarcoidosis was 7.93 months (95% CI 1.21 to 14.64 months), a similar range to delays described for other chronic inflammatory diseases, including inflammatory bowel disease [52]. The overall sample pool of this study consisted of more males than females (54.7% vs. 45.3%). No difference in delay in diagnosis was found between males and females based on the analysis conducted on case reports. The high number of single-person case studies on misdiagnosis attests to the size of the diagnostic challenge for the clinician. Several factors may influence diagnostic delay of sarcoidosis, including the clinical characteristics of the condition, prevalence, different types/presentation of the condition, clinicians’ and patients’ awareness of the condition, and the availability of diagnostic tests.

The present review found complex and rare features of sarcoidosis, healthcare factors, and patient-centred factors may contribute to diagnostic delay in all types of sarcoidosis. In some studies included in the review, associations were found between pulmonary symptoms and higher Scadding scores, and prolonged diagnostic delay of sarcoidosis. The presence of pulmonary symptoms that may be attributable to various health conditions (common flu, pneumonia, bronchitis, asthma, emphysema, and lung cancer) create challenges for healthcare providers working to narrow down the health condition and differentiate between possible causes of pulmonary symptoms. In these cases, healthcare providers may first choose to investigate more common causes of pulmonary symptoms and pursue a diagnostic approach that excludes the most common causes through minimal testing, which is cost effective.

The review also revealed healthcare factors (exclusion diagnosis, challenges with obtaining a biopsy and lack of standard procedure to distinguish sarcoidosis) may lead to diagnostic delay. Difficulties with access to medical resources needed to conduct a biopsy (availability of clinicians and medical facilities) may cause delay in the definitive diagnosis of sarcoidosis through extending the time between suspicion and confirmation of diagnosis. Identifying the difference between suspicion and confirmation of diagnosis can provide further insights into the depth of the impact on diagnostic delay associated with healthcare factors.

Diagnostic delay due to misdiagnosis of tuberculosis was also identified in this review, highlighting the similarity of the two conditions and that differentiating between them is crucial for initiating the correct treatment, as treatment of sarcoidosis involves immunosuppression. In countries with a high prevalence of tuberculosis, it is understandable that clinicians may initially suspect tuberculosis. A misdiagnosis of tuberculosis has implications for the individual, their families and carers, and the use of medical resources, signalling the need for a careful and methodical approach in diagnosis. Once a clinician has made a diagnosis, it is natural to attribute the constellation of symptoms and signs of a rare disease to the identified cause (misdiagnosed condition), until clear evidence arises to disprove the current diagnosis.

Both acute and chronic presentation of sarcoidosis may influence the diagnostic delay. Acute sarcoidosis may follow acute onset with more typical features and radiological findings (hilar adenopathy in chest x-ray). Chronic sarcoidosis has insidious onset and may mimic other disorders (signs and symptoms from multiple systems); therefore, chronic sarcoidosis may present additional challenges for diagnosis of sarcoidosis. The present review did not study the difference in diagnostic delay between acute and chronic sarcoidosis due to limited data. Analyses of sarcoidosis location and factors related to diagnostic delay revealed similar findings, in which most reported factors were linked to complex and rare features, regardless of the location of sarcoidosis. Raising clinicians’ awareness of the complex clinical presentations of all types of sarcoidosis, including rare presentations, may assist in expediting diagnosis.

While none of the included studies used quantitative methods to examine outcomes of diagnostic delay, we used meta-aggregation to extract and examine outcomes described in case reports and case series which revealed incorrect diagnosis, incorrect treatment, and complications/progression of the condition as outcomes of diagnostic delay of sarcoidosis. This accords with findings from a recent review of diagnostic delay in myositis where outcomes including misdiagnoses, progression of symptoms, incorrect treatment, and early discharge were reported [53]. These outcomes align with people’s experiences of diagnostic delay recently described [54], signalling the need for improved awareness of sarcoidosis and a better understanding of its diagnosis and treatment.

As in our previous study examining diagnostic delay of myositis, where we did not find any studies examining people’s experiences of diagnostic delay [53], we did not find research examining experiences of diagnostic delay of sarcoidosis. We believe that further exploration of people’s experiences from symptom onset until diagnosis may assist in understanding these experiences and factors that may impact and influence diagnosis and its delay in sarcoidosis. This information may then be used to inform strategies aimed at reducing the undiagnosed period, including raising awareness and the development of clinical reasoning tools to distinguish when clinicians might consider re-evaluation of an existing diagnosis and the presence of a rare disease.

Despite the lack of studies examining people with sarcoidosis’ experiences of diagnostic delay, a recent commentary describes people with sarcoidosis’ experiences of misdiagnoses [54]. One person described frustration at ‘being dismissed’ and not listened to by their clinician, an experience that has also been described by people with multiple sclerosis seeking a diagnosis [55]. All of those interviewed for the article highlighted ongoing pain and discomfort from symptoms pre- and post-diagnosis as greatly impacting their lives, aligning with evidence of the negative impact that sarcoidosis has on people’s quality of life [56].

Diagnostic delay can create a sense of uncertainty and, in many cases, escalating symptoms, as found in research examining people’s experiences with multiple sclerosis [55], placing them in a stressful state of ‘not knowing”. Delayed diagnosis of childhood illnesses has consequences for both children and their families, including anxiety, frustration and stress, and fear of future reproduction due to ill-defined genetic risk [57]. Hospitalisation and surgical interventions related to rare diseases are more frequent among people who experience a delayed diagnosis [58]. Research examining experiences of hereditary angioedema found that inappropriate treatments were ineffective and at times, exacerbated the underlying condition [59]. For some patients, symptoms were attributed to psychological reasons and due to this, some stopped seeking medical care despite experiencing severe symptoms [59]. Attribution of rare disease symptoms to psychological or psychiatric reasons, and treatment in line with this is not uncommon; [57–59] however the impact of a rare disease on individuals’ mental health has important implications for the treatment and care of people with these health conditions [59].

Delay in diagnosis of sarcoidosis can cause impaired physical function, pain, reduced capacity to work, and strain on personal relationships, leading to a reduction in quality of life and the ability to engage in pleasurable activities, which in turn can have negative emotional consequences that impact wellbeing [60]. A survey of the treatment priorities of people with sarcoidosis found that they most valued quality of life and functionality and concluded that psychological support was key to their wellbeing [61]. Unfortunately, being able to discuss issues and concerns about sarcoidosis with clinician(s) cannot be realised until a diagnosis is received.

## Conclusion

There is a paucity of evidence about the patient experience of diagnostic delay in sarcoidosis and factors related to this. Diagnosis of sarcoidosis can take a long time, during which the impacts on the lives of people living with sarcoidosis can be substantial, including receiving incorrect diagnoses and treatment, and suffering unfavourable outcomes. Further studies examining factors that contribute to diagnostic delay in sarcoidosis, and people’s experiences from symptom onset to diagnosis, are crucial in determining target areas for clinicians, policy-makers and consumer advocacy groups. With this further knowledge, we may develop strategies, training activities and awareness-raising programs that expedite diagnosis and improve outcomes for people living with sarcoidosis.

## Strengths and limitation

The main strength of this review is inclusion of the current evidence of diagnostic delay in all types of studies (including qualitative and quantitative studies) which provided clear insight into the status of diagnostic delay, its factors, and consequences. This systematic review identified a lack of qualitative studies examining patients’ experience of diagnostic delay in sarcoidosis. The main limitation of the present systematic review is the low number of study samples used in pooling of the diagnostic delay (*n* = 124 over 5 studies). The lack of available data on health specialists, clinics, acute or chronic presentation of sarcoidosis, and the period between suspected and confirmed diagnosis limited the possibility of analysing the difference in diagnostic delay in various settings. Lastly, the analysis of case reports may reflect features of chronic sarcoidosis with complex features due to publication bias- tendency to publish rare and interesting cases.

### Electronic supplementary material

Below is the link to the electronic supplementary material.

Supplementary Material 1

Supplementary Material 2

Supplementary Material 3

Supplementary Material 4

## Data Availability

All data relevant to the study is available in the supplementary materials. A detailed extracted data table can be accessed via figtree repository (via DOI: 10.6084/m9.figshare.24431275).
