# Supplementary material for: Diagnostic delay of sarcoidosis: an integrated systematic review
Source: Orphanet J Rare Dis. 2024 Apr 11;19:156. doi: 10.1186/s13023-024-03152-7 (PMC11010435; doi:10.1186/s13023-024-03152-7)
Supplement: Supplementary file 2 — Supplementary Material 2 [file 13023_2024_3152_MOESM2_ESM.docx]

| **Supplementary table 1. Data extraction summary of selected studies categorized according to study designs based on MMAT algorithm** | | | | | | | | | | | | |
| --- | --- | --- | --- | --- | --- | --- | --- | --- | --- | --- | --- | --- |
| **Author** | **Country** | **Population (n)** | **Mean age (year)** | **Organ involvement** | **Health system** | **Diagnostic approach** | **Mean delay**  **(months)** | **Mean delay SD⁂ (months)** | **Initial/presenting symptoms (a) and main symptom (b)** | **Symptom that changed the diagnosis** | **Factors related to delayed diagnosis** | **Experience/Outcomes of diagnostic delay** |
| **1. Non-comparative descriptive study including case reports, case series, survey and descriptive cross-sectional studies** | | | | | | | | | | | | |
| **a. Case reports** | | | | | | | | | | | | |
| Darugar et al., 2011 | Europe | 1 | 26 | Systemic or mixed sarcoidosis | non-gatekeeper | Clinical features and biopsy | 0.5 | NR | a. Blurred vision and black spots in his left eye  b. NR | NR | 1. Broad clinical features and differential diagnosis (Clinical presentation is multisystemic and can be confusing) | NR |
| Froehner et al., 2016 | Europe | 1 | 60 | Extrapulmonary sarcoidosis | gatekeeper | Clinical features and biopsy | 6 | NR | a. Re-current fever and a swollen left-sided kidney  b. NR | NR | 1. Broad clinical features and differential diagnosis (similar features to interstitial nephritis may cause difficulty to diagnose) | 1. Incorrect diagnosis (diagnosed as xanthogranulomatous pyelonephritis)  2. Incorrect treatment (nephrectomy was performed) |
| Ghafoor et al., 2014 | USA | 1 | 69 | Extrapulmonary sarcoidosis | non-gatekeeper | Clinical features and biopsy | 44 | NR | a. Persistent worsening hypertension  b. NR | Worsening hypertension, renal function decline and hypercalcemia | 1. Broad clinical features and differential diagnosis (differentiating between renal diseases in the setting of other underlying co‑morbidities can be challenging) | 1. Incorrect diagnosis (diagnosed as monoclonal gammopathy of undetermined significance)  2. Complication/progression of symptoms (progressed into renal failure and ended up on dialysis) |
| Ghorpade et al., 1996 | Asia | 1 | 50 | Extrapulmonary sarcoidosis | non-gatekeeper | Clinical features and biopsy | 2 | NR | a. A symptomatic reddish skin lesion  b. NR | NR | 1. Broad clinical features and differential diagnosis (cutaneous sarcoidosis can be easily mistaken for lupus vulgaris or leprosy)  2. Rare presentation of sarcoidosis (presenting with skin lesions is rare in sarcoidosis) | NR |
| Ho et al., 2019 | USA | 1 | 55 | Extrapulmonary sarcoidosis | non-gatekeeper | Clinical features and biopsy | 2 | NR | a. Joint pain in the shoulders, wrist, elbows, decreased appetite, weight loss, pruritic skin rash, fatigue, and chills  b. NR | NR | 1. Unawareness and rarity of sarcoidosis (sarcoidosis is rare in Hispanics) | NR |
| Jaster et al., 1997 | USA | 1 | 41 | Extrapulmonary sarcoidosis | non-gatekeeper | Clinical features and biopsy | 18 | NR | a. Clumsiness in hands and difficulty walking  b. NR | NR | 1. Unawareness and rarity of sarcoidosis (spinal sarcoidosis is rare) | NR |
| Lee et al., 2010 | Asia | 1 | 32 | Systemic or mixed sarcoidosis | non-gatekeeper | Clinical features and biopsy | 59 | NR | a. Blurred vision, neck mass, jaw pain and sialadenitis  b. NR | Poor oral intake and strabismus and no response to tuberculosis treatment | 1. Challenges with diagnostic approach (diagnosis of sarcoidosis cannot be made with 100% certainty) | 1. Incorrect diagnosis (tuberculous lymphadenitis)  2. Incorrect treatment (various anti-tuberculous agents) |
| Mehta et al., 2022 | USA | 1 | 49 | Extrapulmonary sarcoidosis | non-gatekeeper | Clinical features and biopsy | 8.5 | NR | a. Bilateral lower extremity weakness, left sided hearing loss, urinary urge incontinence, and gait instability  b. NR | History of sarcoidosis in immediate family member | 1. Broad clinical features and differential diagnosis (hypertensive hemorrhage, cerebral amyloid angiopathy, another CNS vasculitis, or systemic vasculitis with secondary CNS involvement can present with same symptoms as this case)  2. Rare presentation of sarcoidosis (presenting with intracranial haemorrhage in neurosarcoidosis is exceedingly rare)  3. Unawareness and rarity of sarcoidosis (underrepresentation of neurosarcoidosis and haemorrhages) | 1. Complication/progression of symptoms (experienced generalized convulsive seizure) |
| Meyer et al., 2017 | Europe | 1 | 52 | Extrapulmonary sarcoidosis | non-gatekeeper | Clinical features and biopsy | 48 | NR | a. Painful arm cramps  b. NR | Recurrent arm and leg pains with weakness in the extremities | 1. Unawareness and rarity of sarcoidosis (skeletal muscle sarcoidosis is uncommon) | 1 Complication/progression of symptoms (reduced general health and a diffused weakness of extremities) |
| Noiles et al., 2013 | Canada | 1 | 62 | Extrapulmonary sarcoidosis | gatekeeper | Clinical features and biopsy | Several years | NR | a. Painful rash on arms  b. NR | Skin rash worsened and turned into painful red ulcers | 1. Rare presentation of sarcoidosis (ulcerative lesions are rare presentation of cutaneous sarcoidosis) | 1. Incorrect diagnosis (diagnosed as deep tissue infection)  2. Incorrect treatment (antibiotics and antifungal agents)  3. Complication/progression of symptoms (the skin ulcers were secondarily infected) |
| Papaetis et al., 2008 | Europe | 1 | 67 | Pulmonary sarcoidosis | non-gatekeeper | Clinical features and biopsy | 96 | NR | a. Lymphadenopathy, dry cough  b. NR | Persistent fever, shortness of breath and dry cough for a month | 1. Co-existing diseases or comorbidities (diagnostic dilemma that may occur when tuberculosis and sarcoidosis co-exist)  2. Patient centred (she did not agree to a biopsy) | 1. Incorrect diagnosis (diagnosed as bronchitis)  2. Incorrect treatment (various antibiotics)  3. Complication/progression of symptoms (increased dyspnoea and oxygen therapy needed) |
| Plit 1983 | Asia | 1 | 27 | Pulmonary sarcoidosis | non-gatekeeper | Clinical features and biopsy | 18 | NR | a. Persistent productive cough, chest pain, dyspnoea, weight loss, night sweats, deteriorating vision and eye pain  b. NR | No resolution to TB treatment | 1. Rare presentation of sarcoidosis (atypical presentation of sarcoidosis) | 1. Incorrect diagnosis (diagnosed as pulmonary tuberculosis)  2. Incorrect treatment (anti-tuberculous agents) |
| Thomas et al., 2021 | USA | 1 | 48 | Pulmonary sarcoidosis | non-gatekeeper | Clinical features and biopsy | 0.5 | NR | a. Dry cough, fever, red conjunctiva, chills, malaise, myalgias, weight loss and generalized weakness.  b. NR | Persistent cough and other symptoms | 1. Broad clinical features and differential diagnosis (clinical symptoms of sarcoidosis varies)  2. Rare presentation of sarcoidosis (atypical presentation of sarcoidosis) | 1. Incorrect diagnosis (diagnosed as respiratory infection)  2. Incorrect treatment (various antibiotics) |
| van Rooijen et al., 2011 | Europe | 1 | 30 | Systemic or mixed sarcoidosis | gatekeeper | Clinical features and biopsy | 1.4 |  | a. Progressive nausea, vomiting, dizziness, diplopia and headache  b. NR | Intolerable headache, nausea, vomiting | 1. Broad clinical features and differential diagnosis (many internal illnesses can cause hydrocephalus like this case of neurosarcoidosis)  2. Rare presentation of sarcoidosis (hydrocephalus is a rare presentation of sarcoidosis)  3. Challenges with diagnostic approach and tools (diagnosis of neurosarcoidosis is based on exclusion of other possible causes and biopsy is  not always feasible) | 1. Complication/progression of symptoms (intolerable headache, vomiting and blurred vision) |
| Viswanath et al., 2019 | Asia | 1 | 50 | Extrapulmonary sarcoidosis | non-gatekeeper | Clinical features and biopsy | 0.25 | NR | a. Forehead swelling  b. NR | NR | 1. Co-existing diseases or comorbidities (breast cancer and sarcoidosis can present either at the same time or in sequence) | 1. Incorrect diagnosis (diagnosed as subcutaneous metastatic deposit) |
| **b. Case series** | | | | | | | | | | | | |
| Al-Mayouf 2006 | Asia | 8 | 9.3 | NR⁑ | gatekeeper | Clinical features and biopsy | 6 | 4.03 | a. NR  b. Fever, eye involvement, lymphadenopathy | NR | NR | NR |
| Fergie et al., 1999 | Europe | 8 | 44 | Systemic or mixed sarcoidosis | gatekeeper | Clinical features and biopsy | 5 | 5.92 | a. Nasal obstruction (7/8 cases)  epiphora (3/8 cases) nasal discharge (3/8 cases)  epistaxis (1/8 cases), anosmia (1/8 cases)  b. Nasal obstruction is the most common symptom | NR | 1. Broad clinical features and differential diagnosis (there is no single pathognomonic symptom, nasal sarcoidosis’ symptoms are varied and non-specific)  2. Rare presentation of sarcoidosis (As presenting with nasal symptoms are uncommon, occurring in one per cent of patients with sarcoidosis) | NR |
| Guleria et al., 2006 | Asia | 3 | 40.7 | Extrapulmonary sarcoidosis | non-gatekeeper | Clinical features and biopsy | Case 1- 1.5  Case 2 – 18  Case 3 – 6  Case 4- NR  Mean – 8.5 | NR | a. Case 1- Progressive dyspnoea, palpitations  Case 2- dyspnoea and fatigue  Case 3- Generalized body aches, dyspnoea  b. NR | Case 1- Increasing dyspnoea and haemoptysis  Case 2- Persistent tachycardia  Case 3- NR | 1. Broad clinical features and differential diagnosis (diverse symptoms of myocardial sarcoidosis)  2. Unawareness and rarity of sarcoidosis (sarcoidosis is an uncommon disease entity in India and underrepresentation of sarcoidosis in developing countries)  3. Challenges with diagnostic approach and tools (cardiac sarcoidosis diagnosis is made by endomyocardial biopsy which is only positive in 25-50% of patients with cardiac sarcoidosis and and this case may report to different specialists) | 1. Incorrect diagnosis (diagnosed as: case 1- QRS tachycardia, case 2- complete heart block)  2. Incorrect treatment (case 1- amiodarone, case 2- heart implant)  3. Complication/progression of symptoms (in case 3 dyspnoea and haemoptysis increased) |
| Judson et al., 2007 | USA | 2 | 37 | Pulmonary sarcoidosis | non-gatekeeper | Clinical features and biopsy | Case 1- 15  Case 2- 72  Mean – 43.5 | NR | a. Case 1-  Intermittent fever, fatigue, night sweats and weight loss  Case 2- Dyspnoea and cough  b. NR | Case 1- No resolution of symptoms despite treatment  Case 2- No resolution of symptoms with treatment and persistence of symptoms (dyspnoea) | NR | NR |
| Scott et al., 2010 | USA | 8 | NR | Extrapulmonary sarcoidosis | non-gatekeeper | Clinical features and biopsy | NR | NR | a. NR  b. NR | NR | 1. Broad clinical features and differential diagnosis (neurosarcoidosis can have tendency to present indistinguishably from multiple sclerosis) | 1. Incorrect diagnosis (diagnosed as multiple sclerosis) |
| **c. Survey** | | | | | | | | | | | | |
| Kirsten et al., 1995 | Europe | 651 | NR | NR | gatekeeper | NR | 25 | NR | a. NR  b. NR | NR | 1. Challenges with diagnostic approach and tools (insufficient use of bronchic techniques and diagnosis based on chest x ray only) | 1. Incorrect diagnosis (as TB, lung cancer, rheumatic fever, Hodgkin's disease, pneumonia, acting (simulants 2%)) |
| Okumus et al., 2011 | Asia | 293 | 44 | NR | gatekeeper | Clinical features and laboratory features | NR | NR | a. skin lesions (erythema nodosum), arthralgia, back pain, mass in the neck, ocular symptoms  b. NR | NR | NR | NR |
| **d. Descriptive cross-sectional study** | | | | | | | | | | | | |
| Leclerc et al., 2003 | Europe | 28 | NR | NR | non-gatekeeper | Clinical features and biopsy | 6.25 | 3.72 | a. NR  b. NR | NR | NR | NR |
| Send et al., 2019 | Europe | 13 | 48.8 | Extrapulmonary sarcoidosis | non-gatekeeper | Clinical features and biopsy | 8.61 | 6.4 | a. NR  b. Sinusitis, nasal breathing disorder, rhinorrhea, anosmina, and throat pain or swelling, epiphora | NR | 1. Broad clinical features and differential diagnosis (difficulty to distinguish between sarcoidosis sinusitis and other acute sinusitis)  2. Challenges with diagnostic approach and tools (no standard procedure to distinguish sarcoidosis related sinusitis from others) | NR |
| **1. Comparative studies including cohort, case-control studies and analytical cross-sectional study** | | | | | | | | | | | | |
| **a. Analytical cross-sectional study** | | | | | | | | | | | | |
| Bolletta et al., 2020 | Europe | 67 | 55 | Systemic or mixed sarcoidosis | gatekeeper | IWOS 2017 criteria† | 23 | 35 | a. NR  b. NR | NR | 1. Challenges with diagnostic approach and tools (Limited number of patients with lymph nodes amenable to biopsy)  2. Rare presentation of sarcoidosis (The lack of systemic alteration is a major issue for clinicians when trying to diagnose ocular sarcoidosis) | NR |
| Hoogendoorn et al., 2020 | Europe | 15 | 50.7 | Extrapulmonary sarcoidosis | gatekeeper | Clinical features and biopsy | NR | NR | a. NR  b. NR | NR | 1. Broad clinical features and differential diagnosis (cardiac sarcoidosis can mimic other cardiac conditions)  2. Challenges with diagnostic approach and tools (despite use of EMB and F-FDG-PET, none of these two techniques were used in the late diagnosis group) | 1. Complication/progression of symptoms (high irreversible deterioration of cardiac function 6/10)  2. Mortality (high mortality in late diagnosis group 5/10) |
| Judson et al., 2003 ‡ | USA | 189 | NR | NR | NR | Clinical features and biopsy | NR | NR | a. Pulmonary symptoms, skin symptoms, systemic symptoms (fever, malaise, night sweats)  eye, musculoskeletal and abdominal symptoms  b. NR | NR | 1. The presence of pulmonary symptoms were associated with a prolonged time (<6-month vs >6 months) from both onset of symptoms until diagnosis  2. The presence of skin symptoms (usually a skin lesion) was associated with a shorter time (<6 months vs >6 months) to diagnose sarcoidosis from the onset of symptoms  3. Scadding stage and time until diagnosis was related. Patients with stage 0 or I on chest radiographs were more likely to have been diagnosed within 6 months while stage IV patients were least likely to have been diagnosed within 6 months) | NR |
| Kobak et al., 2020 | Asia | 131 | NR | NR | gatekeeper | Clinical features and biopsy | NR | NR | a. NR  b. general symptoms such as fatigue weight loss elderly onset while more classical symptoms such as Erythema nodosum and Lofgren syndrome in young | NR | NR | NR |
| Rodrigues et al., 2013 ‡ | South America | 100 | 47.6 | NR | gatekeeper | Clinical features and biopsy | NR | NR | a. NR  b. Systemic symptoms such as weight loss, night sweats, fever  and pulmonary symptoms such as dyspnea | NR | People in longer diagnostic period (>6 months) were more likely to have been misdiagnosed and treated for tuberculosis | 1. Complication/progression of symptoms (In the delayed diagnosis group, FVC was lower) |
|  |  |  |  |  |  |  |  |  |  |  |  |  |
| ^⁂^SD- Standard deviation  † IWOS 2007- The International Workshop for Ocular Sarcoidosis 2007 criteria  ⁑ NR- Not reported - data that has not been reported in the original study was described as NR  ‡ Studies that used statistical method to compare, measure or explore the link between diagnostic delay and possible factors in the one or more groups | | | | | | | | | | | | |
