# Supplementary material for: Diagnostic delay of sarcoidosis: an integrated systematic review
Source: Orphanet J Rare Dis. 2024 Apr 11;19:156. doi: 10.1186/s13023-024-03152-7 (PMC11010435; doi:10.1186/s13023-024-03152-7)

**Supplementary tables and figures of Diagnostic delay of sarcoidosis: an integrated systematic review**

| Supplementary table 2. Quality assessment using Mixed Methods Appraisal Tool results | | | | | | | | | |
| --- | --- | --- | --- | --- | --- | --- | --- | --- | --- |
| **Non-comparative descriptive study including case reports, case series, survey, descriptive cross-sectional, and survey studies (MMAT 4)** | | | | | | | | | |
| Study | Reviewer | Question 1  Is the sampling strategy relevant to address the research question?  (Yes/No/Can’t tell) | Question 2  Is the sample representative of the target population?  (Yes/No/Can’t tell) | Question 3  Are the measurements appropriate?  (Yes/No/Can’t tell) | Question 4  Is the risk of nonresponse bias low?  (Yes/No/Can’t tell) | Question 5  Is the statistical analysis appropriate to answer the research question?  (Yes/No/Can’t tell) | Low Bias | Unclear Bias | High Bias |
| Al-Mayouf 2006 | TN | No | No | Yes | Yes | Yes | 3 | 0 | 2 |
|  | JD | Can’t tell | No | Yes | Yes | Yes | 3 | 1 | 1 |
|  | Consensus | Can’t tell | No | Yes | Yes | Yes | 3 | 0 | 2 |
| Darugar et al., 2011 | TN | Can’t tell | Can’t tell | Yes | Yes | Yes | 3 | 2 | 0 |
| Fergie et al., 1999 | TN | Can’t tell | Yes | Yes | Yes | Yes | 4 | 1 | 0 |
| Froehner et al., 2016 | TN | No | No | Yes | Yes | Yes | 3 | 0 | 2 |
| Ghafoor et al., 2014 | TN | No | No | Yes | Yes | Yes | 3 | 0 | 2 |
| Ghorpade et al., 1996 | TN | No | No | Yes | Yes | Yes | 3 | 0 | 2 |
| Guleria et al., 2006 | TN | No | No | Yes | Yes | Yes | 3 | 0 | 2 |
| Ho et al., 2019 | TN | No | No | Yes | Yes | Yes | 3 | 0 | 2 |
| Jaster et al., 1997 | TN | No | No | Yes | Yes | Yes | 3 | 0 | 2 |
| Judson et al., 2007 | TN | No | No | Yes | Yes | Yes | 3 | 0 | 2 |
| Kirsten et al., 1995 | TN | Yes | Can’t tell | Yes | No | Yes | 3 | 1 | 1 |
| Leclerc et al., 2003 | TN | Yes | Yes | Yes | Yes | Yes | 5 | 0 | 0 |
| Lee et al., 2010 | TN | No | No | Yes | Yes | Yes | 3 | 0 | 2 |
| Mehta et al., 2022 | TN | No | No | Yes | Yes | Yes | 3 | 0 | 2 |
| Meyer et al., 2017 | TN | No | No | Yes | Yes | Yes | 3 | 0 | 2 |
| Noiles et al., 2013 | TN | No | No | Yes | Yes | Yes | 3 | 0 | 2 |
| Papaetis et al., 2008 | TN | No | No | Yes | Yes | Yes | 3 | 0 | 2 |
| Plit 1983 | TN | No | No | Yes | Yes | Yes | 3 | 0 | 2 |
|  | JD | No | No | Yes | Yes | Yes | 3 | 0 | 2 |
|  | Consensus | No | No | Yes | Yes | Yes | 3 | 0 | 2 |
| Send et al., 2019 | TN | Yes | Yes | Yes | Can’t tell | Yes | 4 | 1 | 0 |
|  | AP | Yes | Can’t tell | Yes | Yes | Yes | 4 | 1 | 0 |
|  | Consensus | Yes | Yes | Yes | Yes | Yes | 5 | 0 | 0 |
| Scott et al., 2010 | TN | Yes | Yes | Yes | Yes | Yes | 5 | 0 | 0 |
|  | AP | Yes | Can’t tell | Yes | Yes | Yes | 4 | 1 | 0 |
|  | Consensus | Yes | Yes | Yes | Yes | Yes | 5 | 0 | 0 |
| Thomas et al., 2021 | TN | No | No | Yes | Yes | Yes | 3 | 0 | 2 |
| van Rooijen et al., 2011 | TN | No | No | Yes | Yes | Yes | 3 | 0 | 2 |
| Viswanath et al., 2019 | TN | No | No | Yes | Yes | Yes | 3 | 0 | 2 |
| **Cohort and case-control studies, analytical cross-sectional study (MMAT 3)** | | | | | | | | | |
| Study | Reviewer | Question 1  Are the participants representative of the target population?  (Yes/No/Can’t tell) | Question 2  Are measurements appropriate regarding both the outcome and intervention (or exposure)?  (Yes/No/Can’t tell) | Question 3  Are there complete outcome data?  (Yes/No/Can’t tell) | Question 4  Are the confounders accounted for in the design and analysis?  (Yes/No/Can’t tell) | Question 5  During the study period, is the intervention administered (or exposure occurred) as intended?  (Yes/No/Can’t tell) | Low Bias | Unclear Bias | High Bias |
| Bolleta et al., 2020 | TN | Yes | Yes | Yes | Can’t tell | Can’t tell | 3 | 2 | 0 |
| Hoogendoorn et al., 2020 | TN | Yes | Yes | Yes | No | Can’t tell | 3 | 1 | 1 |
|  | AP | No | Yes | Yes | Can’t tell | Yes | 3 | 1 | 1 |
|  | Consensus | Yes | Yes | Yes | Yes | Yes | 5 | 0 | 0 |
| Judson et al., 2003 | TN | Can’t tell | Yes | Yes | Can’t tell | Yes | 3 | 2 | 0 |
|  | AP | Can’t tell | Yes | Yes | Can’t tell | Yes | 3 | 2 | 0 |
|  | Consensus | Yes | Yes | Yes | Yes | Yes | 5 | 0 | 0 |
| Kobak et al., 2020 | TN | Yes | Yes | Yes | Can’t tell | Yes | 4 | 1 | 0 |
| Okumus et al., 2011 | TN | Yes | Yes | Can’t tell | Can’t tell | Can’t tell | 2 | 3 | 0 |
| Rodrigues et al., 2013 | TN | Yes | Yes | Yes | Can’t tell | Can’t tell | 3 | 2 | 0 |

| **Supplementary table 3. Meta-aggregation of initial symptoms** | | | |
| --- | --- | --- | --- |
| **Author** | **Raw initial symptoms** | **Aggregation** | **Aggregation results** |
| **1. Non-comparative descriptive study including case reports, case series, survey and descriptive cross-sectional studies** | | | |
| **a. Case reports** | | | |
| Darugar et al., 2011 | Blurred vision | Organ specific (eye symptoms) | Organ specific extrapulmonary (2 symptoms) |
|  | Black spots in his left eye | Organ specific (eye symptoms) |  |
| Froehner et al., 2016 | Re-current fever | General symptoms | General symptoms (1 symptom) |
|  | Swollen left-sided kidney | Organ specific (kidney symptoms) | Organ specific extrapulmonary symptoms (3 symptoms) |
| Ghafoor et al., 2014 | Persistent worsening hypertension | Organ specific (cardiac symptoms) |  |
| Ghorpade et al., 1996 | A symptomatic reddish skin lesion | Organ specific (skin symptoms) |  |
| Ho et al., 2019 | Joint pain in the shoulders, wrist, elbows | General symptoms | General symptoms (6 symptoms) |
|  | Decreased appetite | General symptoms |  |
|  | Weight loss | General symptoms |  |
|  | Pruritic skin rash | General symptoms |  |
|  | Fatigue | General symptoms |  |
|  | Chills | General symptoms |  |
| Jaster et al., 1997 | Clumsiness in hands and difficulty walking | Organ specific (nervous system) | Organ specific symptoms extrapulmonary symptoms (10 symptoms) |
| Lee et al., 2010 | Blurred vision, | Organ specific (nervous system and muscle) |  |
|  | Neck mass, | Organ specific (lymph nodes) |  |
|  | Jaw pain and sialadenitis | Organ specific (glands) |  |
| Mehta et al., 2022 | Bilateral lower extremity weakness, | Organ specific (nervous system) |  |
|  | Left sided hearing loss, | Organ specific (nervous system) |  |
|  | Urinary urge incontinence, | Organ specific (nervous system) |  |
|  | Gait instability | Organ specific (nervous system) |  |
| Meyer et al., 2017 | Painful arm cramps | Organ specific (muscle) |  |
| Noiles et al., 2013 | Painful rash on arms | Organ specific (skin) |  |
| Papaetis et al., 2008 | Lymphadenopathy, | Organ specific symptoms (pulmonary symptoms) | Organ specific pulmonary symptoms (5 symptoms) |
|  | Dry cough | Organ specific symptoms (pulmonary symptoms) |  |
| Plit 1983 | Persistent productive cough, | Organ specific symptoms (pulmonary symptoms) |  |
|  | Chest pain, | Organ specific symptoms (pulmonary symptoms) |  |
|  | Dyspnoea, | Organ specific symptoms (pulmonary symptoms) |  |
|  | Weight loss, | General symptoms | General symptoms (2 symptoms) |
|  | Night sweats, | General symptoms |  |
|  | Deteriorating vision | Organ specific symptoms (eye) | Organ specific extrapulmonary symptoms (3 symptoms) |
|  | Eye pain | Organ specific symptoms (eye) |  |
| Thomas et al., 2021 | Red conjunctiva, | Organ specific symptoms (eye) |  |
|  | Dry cough, | Organ specific symptoms (pulmonary symptoms) | Organ specific pulmonary symptoms (1 symptom) |
|  | Chills, | General symptoms | General symptoms (5 symptoms) |
|  | Malaise, | General symptoms |  |
|  | Myalgias, | General symptoms |  |
|  | Weight loss, | General symptoms |  |
|  | Fever, | General symptoms |  |
|  | Generalized weakness | Organ specific (nervous symptom) | Organ specific extrapulmonary symptoms (7 symptoms) |
| van Rooijen et al., 2011 | Progressive nausea, | Organ specific (nervous symptom) |  |
|  | Vomiting, | Organ specific (nervous symptom) |  |
|  | Dizziness, | Organ specific (nervous symptom) |  |
|  | Diplopia | Organ specific (nervous symptom) |  |
|  | Headache | Organ specific (nervous symptom) |  |
| Viswanath et al., 2019 | Forehead swelling | Organ specific (subcutaneous) |  |
| **b. Case series** | | | |
| Al-Mayouf 2006 | NR⁑ | NR | NR |
| Fergie et al., 1999 | Nasal obstruction (7/8 cases) | Organ specific symptoms (nasal symptoms) | Organ specific extrapulmonary symptoms (7 symptoms) |
|  | Epiphora (3/8 cases) | Organ specific symptoms (nasal symptoms) |  |
|  | Nasal discharge (3/8 cases) | Organ specific symptoms (nasal symptoms) |  |
|  | Epistaxis (1/8 cases), | Organ specific symptoms (nasal symptoms) |  |
|  | Anosmia (1/8 cases) | Organ specific symptoms (nasal symptoms) |  |
| Guleria et al., 2006 | Case 1- Progressive dyspnoea, | Organ specific symptoms (cardiac symptoms) |  |
|  | Case 1- palpitations | Organ specific symptoms (cardiac symptoms) |  |
|  | Case 2- dyspnoea | Organ specific symptoms (pulmonary symptoms) | Organ specific symptoms pulmonary symptoms (2 symptoms) |
|  | Case 3- dyspnoea | Organ specific symptoms (pulmonary symptoms) |  |
|  | Case 3- generalized body aches | General symptoms | General symptoms (6 symptoms) |
|  | Case 2- fatigue | General symptoms |  |
| Judson et al., 2007 | Case 1- intermittent fever, | General symptoms |  |
|  | Case 1- fatigue, | General symptoms |  |
|  | Case 1- night sweats | General symptoms |  |
|  | Case 1- weight loss | General symptoms |  |
|  | Case 2- dyspnoea | Organ specific symptoms (pulmonary symptoms) | Organ specific pulmonary symptoms (2 symptom) |
|  | Case 2- cough | Organ specific symptoms (pulmonary symptoms) |  |
| Scott et al., 2010 | NR | | |
| **c. Survey** | | | |
| Kirsten et al., 1995 | NR | | |
| Okumus et al., 2011 | Back pain | General symptoms | General symptoms (2 symptoms) |
|  | Arthralgia | General symptoms |  |
|  | Skin lesions (erythema nodosum) | Organ specific symptoms (skin symptoms) | Organ specific extrapulmonary symptoms (3 symptoms) |
|  | Mass in the neck | Organ specific symptoms (lymph nodes symptoms) |  |
|  | Ocular symptoms | Organ specific symptoms (eye symptoms) |  |
| **d. Descriptive cross-sectional study** | | | |
| Leclerc et al., 2003 | NR | NR |  |
| Send et al., 2019 | Sinusitis, | Organ specific symptoms (nasal symptoms) | Organ specific extrapulmonary symptoms (6 symptoms) |
|  | Nasal breathing disorder, | Organ specific symptoms (nasal symptoms) |  |
|  | Rhinorrhea, | Organ specific symptoms (nasal symptoms) |  |
|  | Anosmina, | Organ specific symptoms (nasal symptoms) |  |
|  | Throat pain/swelling | Organ specific symptoms (throat symptoms) |  |
|  | Epiphora | Organ specific symptoms (nasal symptoms) |  |
| **2.** **Comparative studies including cohort, case-control studies and analytical cross-sectional study** | | | |
| **a. Analytical cross-sectional study** | | | |
| Bolletta et al., 2020 | NR | | |
| Hoogendoorn et al., 2020 | NR | | |
| Judson et al., 2003 | Pulmonary symptoms, | Organ specific symptoms (pulmonary symptoms) | Organ specific pulmonary symptoms (1 symptom) |
|  | Skin symptoms | Organ specific symptoms (skin symptoms) | Organ specific extrapulmonary symptoms (3 symptoms) |
|  | Musculoskeletal | Organ specific symptoms (musculoskeletal symptoms) |  |
|  | Abdominal symptoms | Organ specific symptoms (abdominal symptoms) |  |
|  | Fever | General symptoms | General symptoms (3 symptoms) |
|  | Malaise | General symptoms |  |
|  | Night sweats | General symptoms |  |
| Kobak et al., 2020 | NR | | |
| Rodrigues et al., 2013 | NR | | |
| ⁑ NR- Not reported - data that has not been reported in the original study was described as NR | | | |

| **Supplementary table 4. Meta-aggregation of symptoms that changed the diagnosis** | | | |
| --- | --- | --- | --- |
| **Author** | **Symptom that changed the diagnosis** | **Aggregation** | **Aggregation results** |
| **1. Non-comparative descriptive study including case reports, case series, survey and descriptive cross-sectional studies** | | | |
| **a. Case reports** | | | |
| Darugar et al., 2011 | NR⁑ | | |
| Froehner et al., 2016 | NR | | |
| Ghafoor et al., 2014 | Worsening hypertension, | Worsening symptoms | Worsening symptoms (3 symptoms) |
|  | Renal function decline | Worsening symptoms |  |
|  | and hypercalcemia | Worsening symptoms |  |
| Ghorpade et al., 1996 | NR | | |
| Ho et al., 2019 | NR | | |
| Jaster et al., 1997 | NR | | |
| Lee et al., 2010 | Poor oral intake | New symptom or sign | New symptom or sign (2 symptoms) |
|  | Strabismus | New symptom or sign |  |
|  | No response to treatment | No response to treatment | No response to treatment (1 symptom) |
| Mehta et al., 2022 | History of sarcoidosis in immediate family member | New symptom or sign | New symptom or sign (2 symptoms) |
| Meyer et al., 2017 | Lower extremities weakness | New symptom or sign |  |
|  | Recurrent arm and leg pains | Persistent symptom | Persistent symptom (1 symptoms) |
| Noiles et al., 2013 | Skin rash worsened and turned into painful red ulcers | Worsening symptoms | Worsening symptoms (1 symptom) |
| Papaetis et al., 2008 | Persistent fever, | Persistent symptom | Persistent symptom (3 symptoms) |
|  | Shortness of breath | Persistent symptom |  |
|  | Dry cough for a month | Persistent symptom |  |
| Plit 1983 | No resolution to TB treatment | No response to treatment | No response to treatment (1 symptom) |
| Thomas et al., 2021 | Persistent cough and other symptoms | Persistent symptom | Persistent symptom (1 symptoms) |
| van Rooijen et al., 2011 | Intolerable headache, | New symptom or sign | New symptom or sign (3 symptoms) |
|  | Nausea, | New symptom or sign |  |
|  | Vomiting | New symptom or sign |  |
| Viswanath et al., 2019 | NR | | |
| **b. Case series** | | | |
| Al-Mayouf 2006 | NR | | |
| Fergie et al., 1999 | NR | | |
| Guleria et al., 2006 | Case 1- Increasing dyspnoea | Worsening of symptoms | Worsening of symptoms (2 symptoms) |
|  | Case 1- Increasing haemoptysis | Worsening of symptoms |  |
|  | Case 2- Persistent tachycardia | Persistent symptom | Persistent symptom (1 symptom) |
| Judson et al., 2007 | Case 2- persistence of symptoms (dyspnoea) | Persistent symptom | Persistent symptom (1 symptom) |
|  | Case 2- No resolution of symptoms with treatment | No response to treatment | No response to treatment (2 symptoms) |
|  | Case 1- No resolution of symptoms despite treatment | No response to treatment |  |
| Scott et al., 2010 | NR | | |
| **c. Survey** | | |  |
| Kirsten et al., 1995 | NR  NR | | |
| Okumus et al., 2011 | NR | | |
| **d. Descriptive cross-sectional study** | | | |
| Leclerc et al., 2003 | NR | | |
| Send et al., 2019 | NR | | |
| **2.** **Comparative studies including cohort, case-control studies and analytical cross-sectional study** | | | |
| **a. Analytical cross-sectional study** | | | |
| Bolletta et al., 2020 | NR | | |
| Hoogendoorn et al., 2020 | NR | | |
| Judson et al., 2003 | NR | | |
| Kobak et al., 2020 | NR | | |
| Rodrigues et al., 2013 | NR | | |
| ⁑ NR- Not reported - data that has not been reported in the original study was described as NR | | | |

| **Supplementary table 5. Meta-aggregation of factors** | | | |
| --- | --- | --- | --- |
| **Author** | **Factors mentioned in the study** | **Aggregation** | **Aggregation results** |
| **1. Non-comparative descriptive study including case reports, case series, survey and descriptive cross-sectional studies** | | | |
| **a. Case reports** | | | |
| Darugar et al., 2011 | Clinical presentation is multisystemic and can be confusing | 1. Broad clinical features and differential diagnosis | **Complex and rare features of sarcoidosis (8 factors)** |
| Froehner et al., 2016 | Similar features to interstitial nephritis may cause difficulty to diagnose | 1. Broad clinical features and differential diagnosis |  |
| Ghafoor et al., 2014 | Differentiating between renal diseases in the setting of other underlying co‑morbidities can be challenging | 1. Broad clinical features and differential diagnosis |  |
| Ghorpade et al., 1996 | Cutaneous sarcoidosis can be easily mistaken for lupus vulgaris or leprosy | 1. Broad clinical features and differential diagnosis |  |
|  | Presenting with skin lesions is rare in sarcoidosis | 2. Rare presentation of sarcoidosis |  |
| Ho et al., 2019 | Sarcoidosis is rare in Hispanics | 3. Unawareness and rarity of sarcoidosis |  |
| Jaster et al., 1997 | Spinal sarcoidosis is rare | 3. Unawareness and rarity of sarcoidosis |  |
| Lee et al., 2010 | Hypertensive haemorrhage, cerebral amyloid angiopathy, another CNS vasculitis, or systemic vasculitis with secondary CNS involvement can present with same symptoms as this case | 1. Broad clinical features and differential diagnosis |  |
| Mehta et al., 2022 | Diagnosis of sarcoidosis cannot be made with 100% certainty | 4. Challenges with diagnostic approach | **Health care factors (1 factor)** |
|  | Presenting with intracranial haemorrhage in neurosarcoidosis is exceedingly rare | 5. Rare presentation of sarcoidosis | **Complex and rare features of sarcoidosis (5 factors)** |
|  | Underrepresentation of neurosarcoidosis and haemorrhages | 3. Unawareness and rarity of sarcoidosis |  |
| Meyer et al., 2017 | Skeletal muscle sarcoidosis is uncommon | 3. Unawareness and rarity of sarcoidosis |  |
| Noiles et al., 2013 | Ulcerative lesions are rare presentation of cutaneous sarcoidosis | 5. Rare presentation of sarcoidosis |  |
| Papaetis et al., 2008 | Diagnostic dilemma that may occur when tuberculosis and sarcoidosis co-exist | 6. Co-existing diseases or comorbidities |  |
|  | She did not agree to a biopsy | 7. Patient centred | **Patient centred factor (1 factor)** |
| Plit 1983 | Atypical presentation of sarcoidosis | 5. Rare presentation of sarcoidosis | **Complex and rare features of sarcoidosis (6 factors)** |
| Thomas et al., 2021 | Clinical symptoms of sarcoidosis vary | 1. Broad clinical features and differential diagnosis |  |
|  | Atypical presentation of sarcoidosis | 5. Rare presentation of sarcoidosis |  |
| van Rooijen et al., 2011 | Many internal illnesses can cause hydrocephalus like this case of neurosarcoidosis | 1. Broad clinical features and differential diagnosis |  |
|  | Hydrocephalus is a rare presentation of sarcoidosis | 5. Rare presentation of sarcoidosis |  |
|  | Breast cancer and sarcoidosis can present either at the same time or in sequence | 6. Co-existing diseases or comorbidities |  |
| Viswanath et al., 2019 | Diagnosis of neurosarcoidosis is based on exclusion of other possible causes and biopsy is not always feasible | 4. Challenges with diagnostic approach and tools | **Health care factors (1 factor)** |
| **b. Case series** | | | |
| Al-Mayouf 2006 |  |  |  |
| Fergie et al., 1999 | There is no single pathognomonic symptom, nasal sarcoidosis’ symptoms are varied and non-specific | 1. Broad clinical features and differential diagnosis | **Complex and rare features of sarcoidosis (4 factors)** |
|  | As presenting with nasal symptoms are uncommon, occurring in one per cent of patients with sarcoidosis | 5. Rare presentation of sarcoidosis |  |
| Guleria et al., 2006 | Diverse symptoms of myocardial sarcoidosis | 1. Broad clinical features and differential diagnosis |  |
|  | Sarcoidosis is an uncommon disease entity in India and underrepresentation of sarcoidosis in developing countries | 3. Unawareness and rarity of sarcoidosis |  |
|  | Cardiac sarcoidosis diagnosis is made by endomyocardial biopsy which is only positive in 25-50% of patients with cardiac sarcoidosis and this case may report to different specialists | 4. Challenges with diagnostic approach and tools | **Health care factors (1 factor)** |
| Judson et al., 2007 | NR⁑ | | |
| Scott et al., 2010 | Neurosarcoidosis can have tendency to present indistinguishably from multiple sclerosis | 1. Broad clinical features and differential diagnosis | **Complex and rare features of sarcoidosis (1 factor)** |
| **c. Survey** | | | |
| Kirsten et al., 1995 | Insufficient use of bronchic techniques and diagnosis based on chest x-ray only | 4. Challenges with diagnostic approach and tools | **Health care factors (1 factor)** |
| Okumus et al., 2011 | NR | | |
| **d. Descriptive cross-sectional study** | | | |
| Leclerc et al., 2003 | NR | | |
| Send et al., 2019 | Difficulty to distinguish between sarcoidosis sinusitis and other acute sinusitis | 1. Broad clinical features and differential diagnosis | **Complex and rare features of sarcoidosis (1 factor)** |
|  | No standard procedure to distinguish sarcoidosis related sinusitis from others | 4. Challenges with diagnostic approach and tools | **Health care factors (1 factor)** |
| **2.** **Comparative studies including cohort, case-control studies, and analytical cross-sectional study** | | | |
| **a. Analytical cross-sectional study** | | | |
| Bolletta et al., 2020 | Limited number of patients with lymph nodes amenable to biopsy | 4. Challenges with diagnostic approach and tools | **Health care factors (1 factor)** |
|  | The lack of systemic alteration is a major issue for clinicians when trying to diagnose ocular sarcoidosis | 5. Rare presentation of sarcoidosis | **Complex and rare features of sarcoidosis (2 factors)** |
| Hoogendoorn et al., 2020 | Cardiac sarcoidosis can mimic other cardiac conditions | 1. Broad clinical features and differential diagnosis |  |
|  | Despite use of EMB and F-FDG-PET, none of these two techniques were used in the late diagnosis group | 5. Health care provider centred | **Health care factors (1 factor)** |
| Kobak et al., 2020 | NR | | |
| ⁑ NR- Not reported - data that has not been reported in the original study was described as NR  ‡ Studies that used statistical method to compare, measure or explore the link between diagnostic delay and possible factors in the one or more groups | | | |

| **Supplementary table 6. Meta-aggregation of factors by sarcoidosis types** | | | |
| --- | --- | --- | --- |
| **Author** | **Factors mentioned in the study** | **Aggregation** | **Aggregation results** |
| **I. Pulmonary sarcoidosis** | | | |
| **1. Non-comparative descriptive study including case reports, case series, survey and descriptive cross-sectional studies** | | | |
| **a. Case reports** | | | |
| Papaetis et al., 2008 | Diagnostic dilemma that may occur when tuberculosis and sarcoidosis co-exist | 6. Co-existing diseases or comorbidities | **Complex and rare features of sarcoidosis (1 factor)** |
|  | She did not agree to a biopsy | 7. Patient centred | **Patient centred factor (1 factor)** |
| Plit 1983 | Atypical presentation of sarcoidosis | 5. Rare presentation of sarcoidosis | **Complex and rare features of sarcoidosis (1 factor)** |
| Thomas et al., 2021 | Clinical symptoms of sarcoidosis vary | 1. Broad clinical features and differential diagnosis | **Complex and rare features of sarcoidosis (2 factors)** |
|  | Atypical presentation of sarcoidosis | 5. Rare presentation of sarcoidosis |  |
| **II. Extrapulmonary sarcoidosis** | | | |
| **1. Non-comparative descriptive study including case reports, case series, survey and descriptive cross-sectional studies** | | | |
| **a. Case reports** | | | |
| Froehner et al., 2016 | Similar features to interstitial nephritis may cause difficulty to diagnose | 1. Broad clinical features and differential diagnosis | **Complex and rare features of sarcoidosis (6 factors)** |
| Ghafoor et al., 2014 | Differentiating between renal diseases in the setting of other underlying co‑morbidities can be challenging | 1. Broad clinical features and differential diagnosis |  |
| Ghorpade et al., 1996 | Cutaneous sarcoidosis can be easily mistaken for lupus vulgaris or leprosy | 1. Broad clinical features and differential diagnosis |  |
|  | Presenting with skin lesions is rare in sarcoidosis | 2. Rare presentation of sarcoidosis |  |
| Ho et al., 2019 | Sarcoidosis is rare in Hispanics | 3. Unawareness and rarity of sarcoidosis |  |
| Jaster et al., 1997 | Spinal sarcoidosis is rare | 3. Unawareness and rarity of sarcoidosis |  |
| Mehta et al., 2022 | Diagnosis of sarcoidosis cannot be made with 100% certainty | 4. Challenges with diagnostic approach | **Health care factors (1 factor)** |
|  | Presenting with intracranial haemorrhage in neurosarcoidosis is exceedingly rare | 5. Rare presentation of sarcoidosis | **Complex and rare features of sarcoidosis (5 factors)** |
|  | Underrepresentation of neurosarcoidosis and haemorrhages | 3. Unawareness and rarity of sarcoidosis |  |
| Meyer et al., 2017 | Skeletal muscle sarcoidosis is uncommon | 3. Unawareness and rarity of sarcoidosis |  |
| Noiles et al., 2013 | Ulcerative lesions are rare presentation of cutaneous sarcoidosis | 5. Rare presentation of sarcoidosis |  |
| Viswanath et al., 2019 | Diagnosis of neurosarcoidosis is based on exclusion of other possible causes and biopsy is  not always feasible | 4. Challenges with diagnostic approach and tools | **Health care factors (1 factor)** |
| **b. Case series** | | | |
| Guleria et al., 2006 | Diverse symptoms of myocardial sarcoidosis | 1. Broad clinical features and differential diagnosis | **Complex and rare features of sarcoidosis (2 factors)** |
|  | Sarcoidosis is an uncommon disease entity in India and underrepresentation of sarcoidosis in developing countries | 3. Unawareness and rarity of sarcoidosis |  |
|  | Cardiac sarcoidosis diagnosis is made by endomyocardial biopsy which is only positive in 25-50% of patients with cardiac sarcoidosis and this case may report to different specialists | 4. Challenges with diagnostic approach and tools | **Health care factors (1 factor)** |
| Scott et al., 2010 | Neurosarcoidosis can have tendency to present indistinguishably from multiple sclerosis | 1. Broad clinical features and differential diagnosis | **Complex and rare features of sarcoidosis (1 factor)** |
| **d. Descriptive cross-sectional study** | | | |
| Send et al., 2019 | Difficulty to distinguish between sarcoidosis sinusitis and other acute sinusitis | 1. Broad clinical features and differential diagnosis | **Complex and rare features of sarcoidosis (1 factor)** |
|  | No standard procedure to distinguish sarcoidosis related sinusitis from others | 4. Challenges with diagnostic approach and tools | **Health care factors (1 factor)** |
| **2.** **Comparative studies including cohort, case-control studies, and analytical cross-sectional study** | | | |
| **a. Analytical cross-sectional study** | | | |
| Hoogendoorn et al., 2020 | Cardiac sarcoidosis can mimic other cardiac conditions | 1. Broad clinical features and differential diagnosis | **Complex and rare features of sarcoidosis (1 factor)** |
|  | Despite use of EMB and F-FDG-PET, none of these two techniques were used in the late diagnosis group | 5. Health care provider centred | **Health care factors (1 factor)** |
| **III. Systemic sarcoidosis** | | | |
| **1. Non-comparative descriptive study including case reports, case series, survey and descriptive cross-sectional studies** | | | |
| **a. Case reports** | | | |
| Darugar et al., 2011 | Clinical presentation is multisystemic and can be confusing | 1. Broad clinical features and differential diagnosis | **Complex and rare features of sarcoidosis (5 factors)** |
| Lee et al., 2010 | Hypertensive haemorrhage, cerebral amyloid angiopathy, another CNS vasculitis, or systemic vasculitis with secondary CNS involvement can present with same symptoms as this case | 1. Broad clinical features and differential diagnosis |  |
| van Rooijen et al., 2011 | Many internal illnesses can cause hydrocephalus like this case of neurosarcoidosis | 1. Broad clinical features and differential diagnosis |  |
|  | Hydrocephalus is a rare presentation of sarcoidosis | 5. Rare presentation of sarcoidosis |  |
|  | Breast cancer and sarcoidosis can present either at the same time or in sequence | 6. Co-existing diseases or comorbidities |  |
| **b. Case series** | | | |
| Fergie et al., 1999 | There is no single pathognomonic symptom, nasal sarcoidosis’ symptoms are varied and non-specific | 1. Broad clinical features and differential diagnosis | **Complex and rare features of sarcoidosis (2 factors)** |
|  | As presenting with nasal symptoms are uncommon, occurring in one per cent of patients with sarcoidosis | 5. Rare presentation of sarcoidosis |  |
| **2.** **Comparative studies including cohort, case-control studies, and analytical cross-sectional study** | | | |
| **a. Analytical cross-sectional study** | | | |
| Bolletta et al., 2020 | Limited number of patients with lymph nodes amenable to biopsy | 4. Challenges with diagnostic approach and tools | **Health care factors (1 factor)** |
|  | The lack of systemic alteration is a major issue for clinicians when trying to diagnose ocular sarcoidosis | 5. Rare presentation of sarcoidosis | **Complex and rare features of sarcoidosis (1 factor)** |
|  | | | |

| **Supplementary table 7. Meta-aggregation results on outcomes related with diagnostic delay** | | | | | |
| --- | --- | --- | --- | --- | --- |
| **Author** | **Factors mentioned in the study** | **Aggregation** | **Aggregation results** | | |
| **1. Non-comparative descriptive study including case reports, case series, survey and descriptive cross-sectional studies** | | | | | |
| **a. Case reports** | | | | | |
| Darugar et al., 2011 | NR⁑ | | | | |
| Froehner et al., 2016 | Nephrectomy was performed | 2. Incorrect treatment | **2. Incorrect treatment (1 case)** | | |
|  | Diagnosed as xanthogranulomatous pyelonephritis | 1. Incorrect diagnosis | **1. Incorrect diagnosis (2 cases)** | | |
| Ghafoor et al., 2014 | Diagnosed as monoclonal gammopathy of undetermined significance | 1. Incorrect diagnosis |  |  |  |
|  | Progressed into renal failure and ended up on dialysis | 3. Complication/progression of symptoms | **3. Complication/progression of symptoms (1 case)** | | |
| Ghorpade et al., 1996 | NR | | | | |
| Ho et al., 2019 | NR | | | | |
| Jaster et al., 1997 | NR | | | | |
| Lee et al., 2010 | Diagnosed as tuberculous lymphadenitis | 1. Incorrect diagnosis | | **1. Incorrect diagnosis (1 case)** | |
|  | Treated with various anti-tuberculous agents | 2. Incorrect treatment | | **2. Incorrect treatment (1 case)** | |
| Mehta et al., 2022 | Experienced generalized convulsive seizure | 3. Complication/progression of symptoms | | **3. Complication/progression of symptoms (2 cases)** | |
| Meyer et al., 2017 | Reduced general health and a diffused weakness of extremities | 3. Complication/progression of symptoms | |  |  |
| Noiles et al., 2013 | Diagnosed as deep tissue infection | 1. Incorrect diagnosis | | **1. Incorrect diagnosis (1 case)** | |
|  | Treated with antibiotics and antifungal agents | 2. Incorrect treatment | | **2. Incorrect treatment (1 case)** | |
|  | Skin ulcers were secondarily infected | 3. Complication/progression of symptoms | | **3. Complication/progression of symptoms (2 cases)** | |
| Papaetis et al., 2008 | Increased dyspnoea and oxygen therapy needed | 3. Complication/progression of symptoms | |  |  |
|  | Treated with various antibiotics | 2. Incorrect treatment | | **2. Incorrect treatment (1 case)** | |
|  | Diagnosed as bronchitis | 1. Incorrect diagnosis | | **1. Incorrect diagnosis (2 cases)** | |
| Plit 1983 | Diagnosed as pulmonary tuberculosis | 1. Incorrect diagnosis | |  |  |
|  | Treated with anti-tuberculous agents | 2. Incorrect treatment | | **2. Incorrect treatment (2 cases)** | |
| Thomas et al., 2021 | Treated with various antibiotics | 2. Incorrect treatment | |  |  |
|  | Diagnosed as respiratory infection | 1. Incorrect diagnosis | | **1. Incorrect diagnosis (1 case)** | |
| van Rooijen et al., 2011 | Developed intolerable headache, vomiting and blurred vision | 3. Complication/progression of symptoms | | **3. Complication/progression of symptoms (1 case)** | |
| Viswanath et al., 2019 | Excised as suspected subcutaneous metastatic deposit (diagnosis on biopsy) | 2. Incorrect treatment | | **2. Incorrect treatment (1 case)** | |
| **b. Case series** |  | | | | |
| Al-Mayouf 2006 | NR | | | | |
| Fergie et al., 1999 | NR | | | | |
| Guleria et al., 2006 | Case 1 diagnosed as QRS tachycardia | 1. Incorrect diagnosis | | **1. Incorrect diagnosis (2 cases)** | |
|  | Case 2- diagnosed as complete heart block | 1. Incorrect diagnosis | |  |  |
|  | Case 1- treated with amiodarone | 1. Incorrect treatment | | **2. Incorrect treatment (2 cases)** | |
|  | Case 2- treated with heart implant | 1. Incorrect treatment | |  |  |
|  | Case 3- dyspnoea and haemoptysis increased | 3. Complication/progression of symptoms | | **3. Complication/progression of symptoms (1 case)** | |
| Judson et al., 2007 | NR | | |  | |
| Scott et al., 2010 | Diagnosed as multiple sclerosis in eight cases | 1. Incorrect diagnosis | | **1. Incorrect diagnosis (1 case)** | |
| **c. Survey** |  |  | |  | |
| Kirsten et al., 1995 | Diagnosed as TB, lung cancer, rheumatic fever, Hodgkin's disease, pneumonia, and acting | 1. Incorrect diagnosis | | **1. Incorrect diagnosis (case number unknown)** | |
| Okumus et al., 2011 | NR | | | | |
| **d. Descriptive cross-sectional study** |  | | | | |
| Leclerc et al., 2003 | NR | | | | |
| Send et al., 2019 | NR | | | | |
| **2.** **Comparative studies including cohort, case-control studies and analytical cross-sectional study** | | | | | |
| **a. Analytical cross-sectional study** | | | | | |
| Bolletta et al., 2020 | NR | | | | |
| Hoogendoorn et al., 2020 | High irreversible deterioration of cardiac function 6/10 | 3. Complication/progression of symptoms | | **3. Complication/progression of symptoms (10 cases)** | |
|  | High mortality in late diagnosis group 5/10 | 4. Mortality | |  |  |
| Judson et al., 2003 | NR | | | | |
| Kobak et al., 2020 | NR | | | | |
| Rodrigues et al., 2013 | In the delayed diagnosis group, FVC was lower | 3. Complication/progression of symptoms | | | **3. Complication/progression of symptoms (case number unknown)** |
| ⁑ NR- Not reported - data that has not been reported in the original study was described as NR | | | | | |

Supplementary figure 1. Colour-enhanced funnel plot demonstrating mean diagnostic delay time for sarcoidosis in five studies


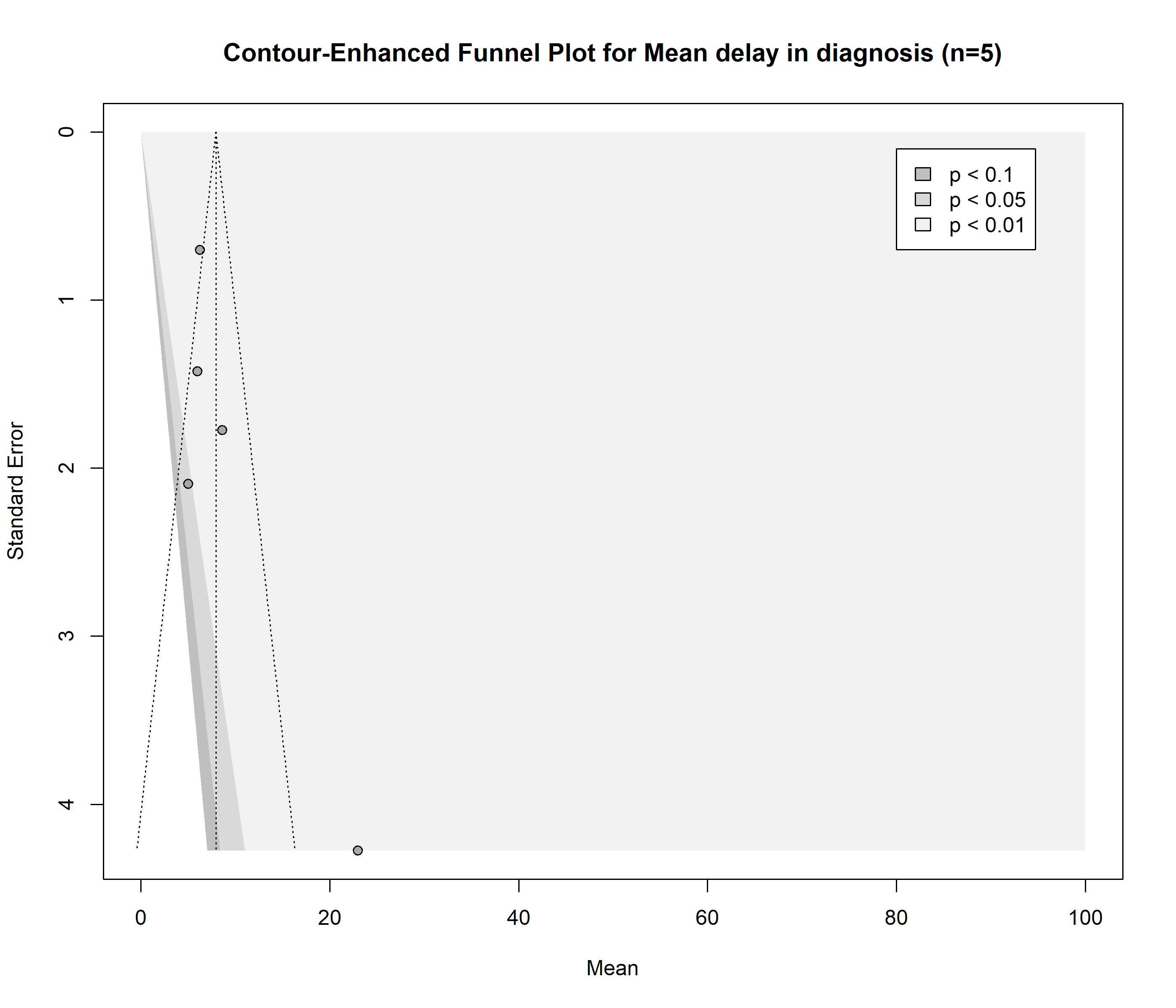


Supplementary figure 2. Sensitivity analysis between SD estimated and non-estimated studies


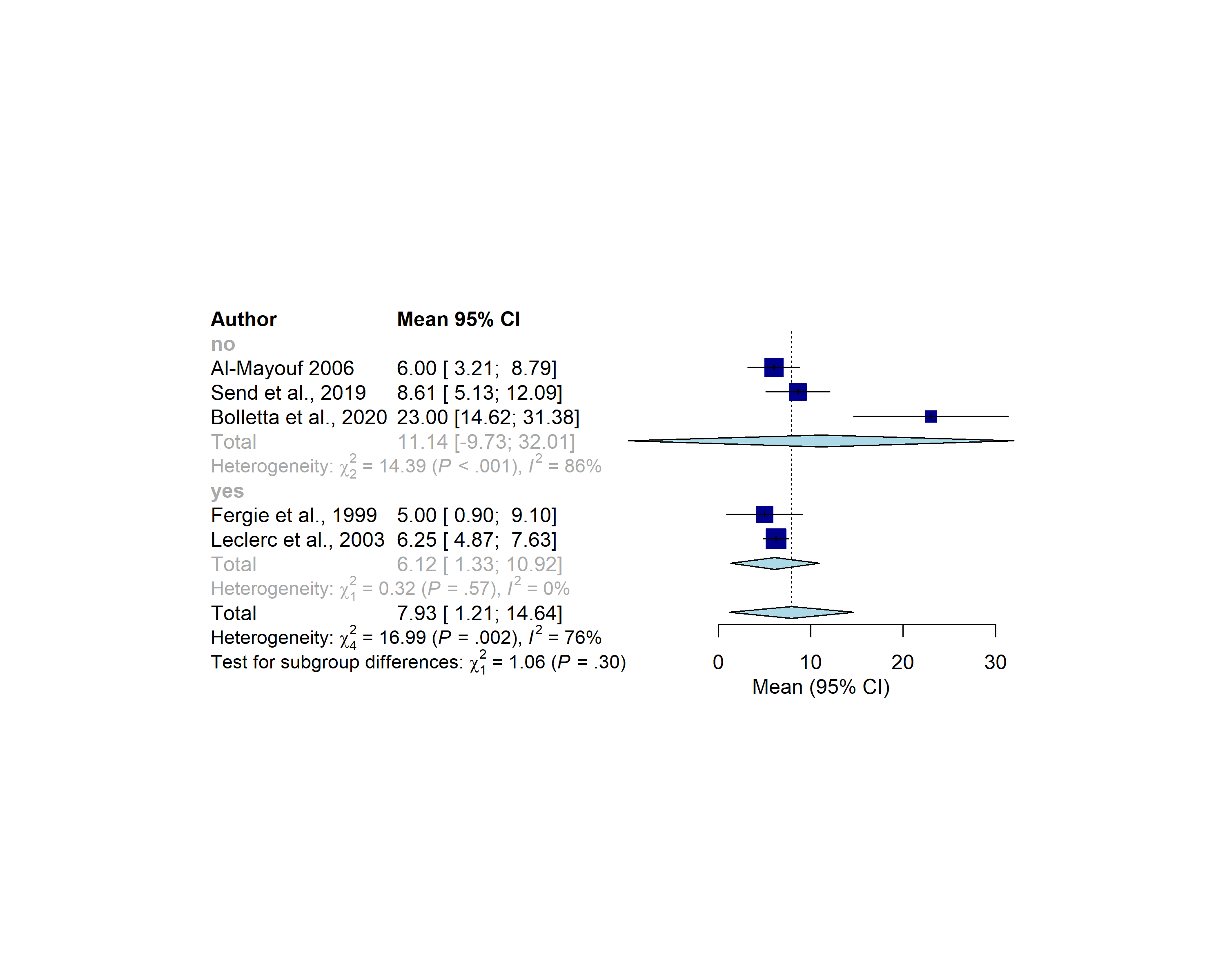


Supplementary figure 3. Subgroup analysis of pooled mean diagnostic delay for sarcoidosis in gatekeeper and non-gatekeeper health systems


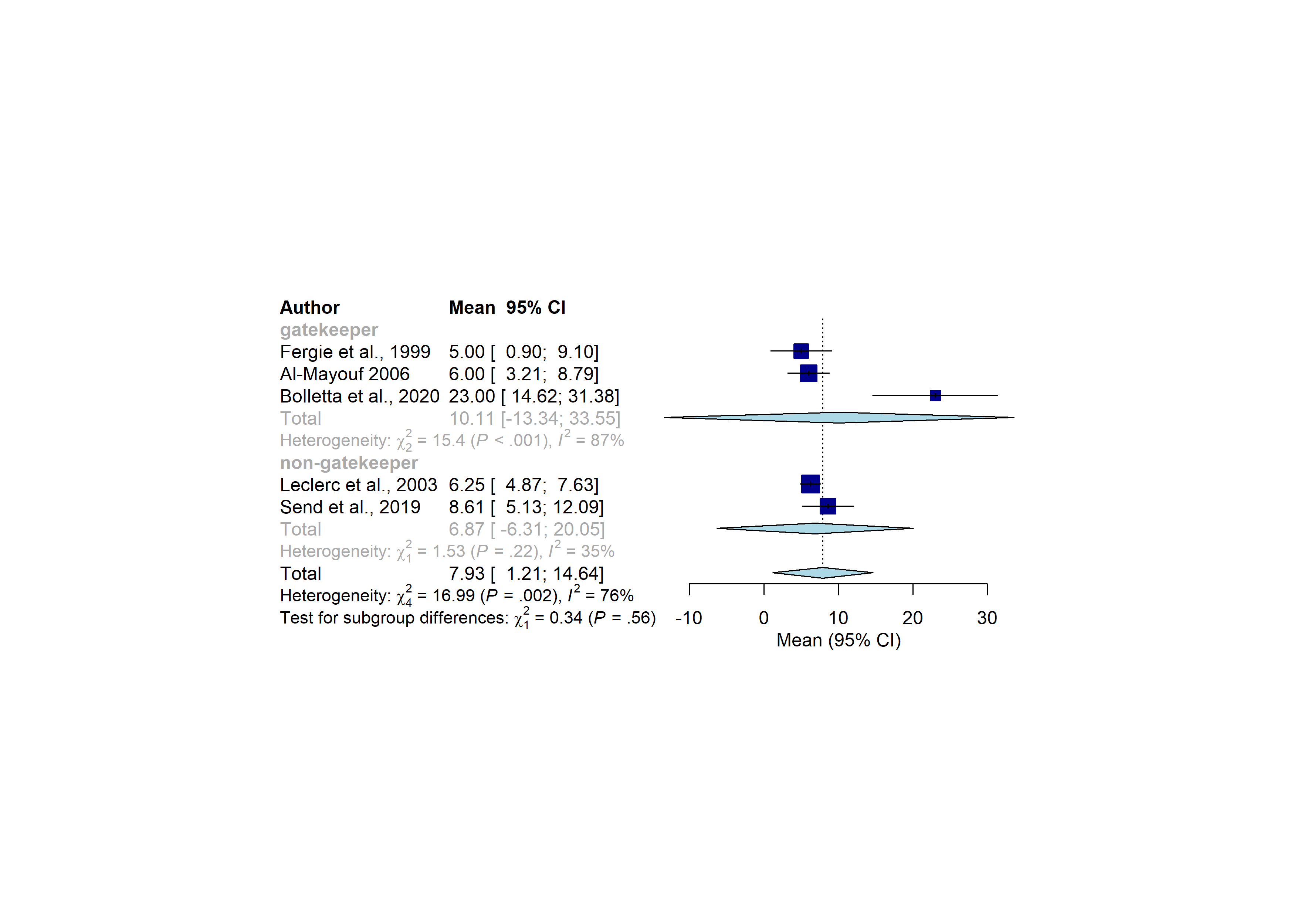


Supplementary figure 4. Subgroup analysis of pooled mean diagnostic delay for sarcoidosis by publication year


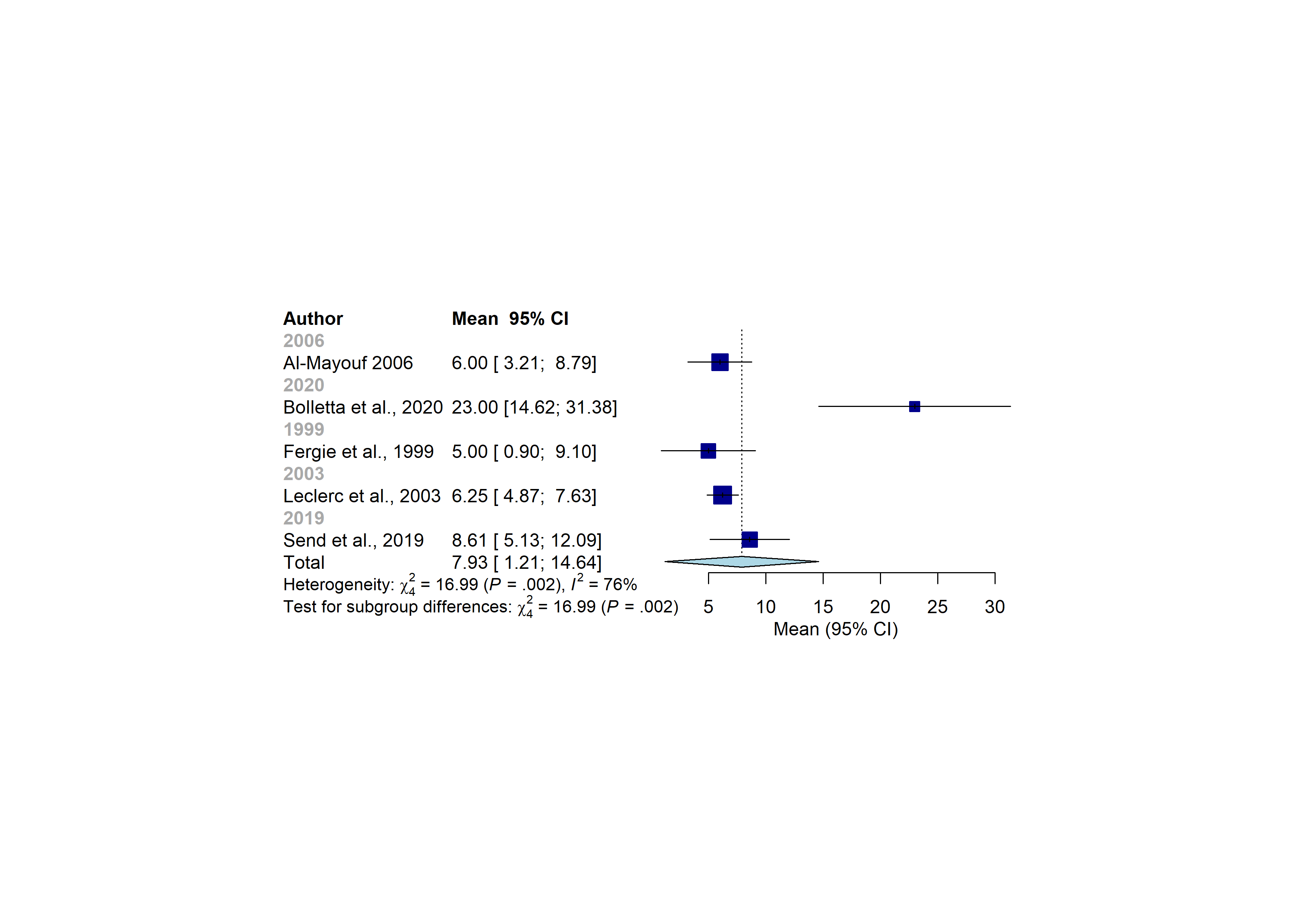


Supplementary figure 5. Meta-aggregation results of symptoms that changed the diagnosis
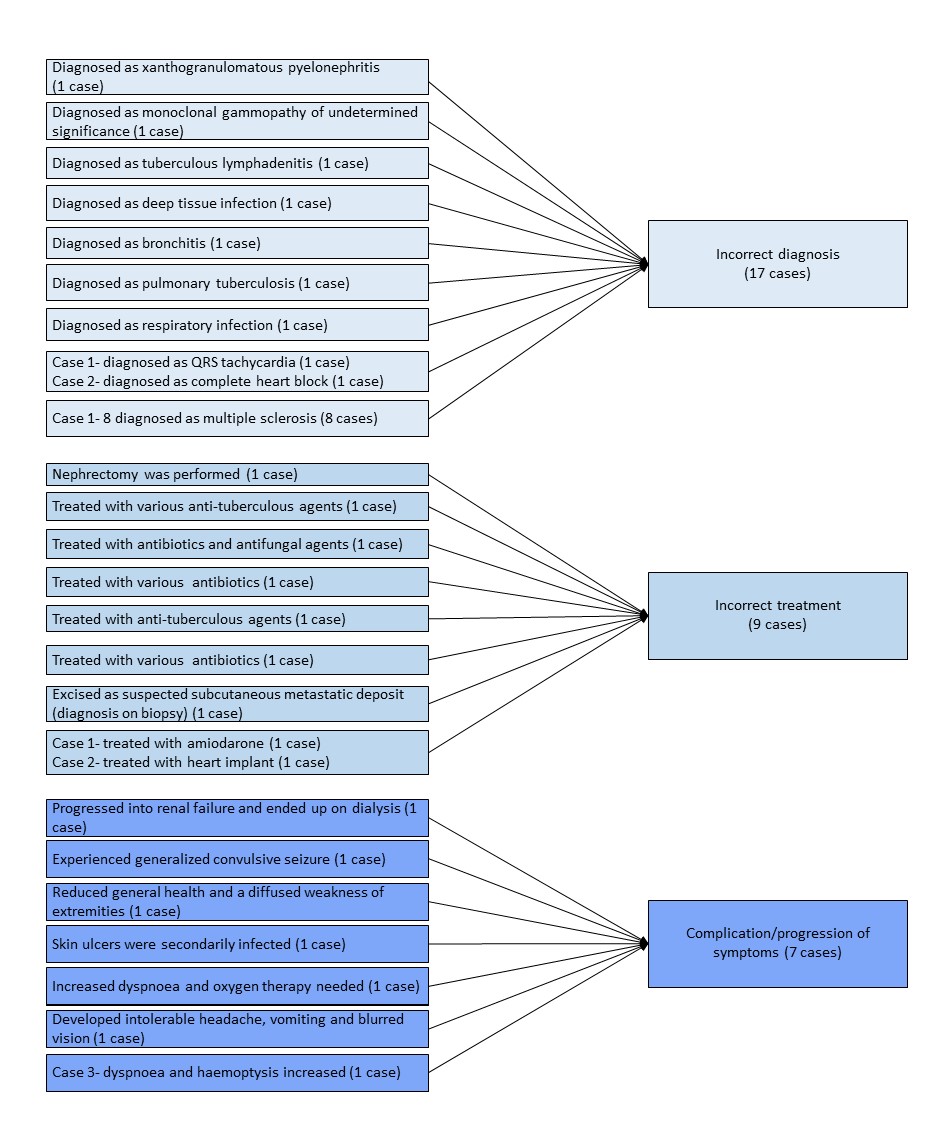

Supplement: Supplementary file 3 — Supplementary Material 3 [file 13023_2024_3152_MOESM3_ESM.docx]
