# Supplementary material for: Diagnostic delay of sarcoidosis: an integrated systematic review
Source: Orphanet J Rare Dis. 2024 Apr 11;19:156. doi: 10.1186/s13023-024-03152-7 (PMC11010435; doi:10.1186/s13023-024-03152-7)
Supplement: Supplementary file 4 — Supplementary Material 4 [file 13023_2024_3152_MOESM4_ESM.docx]

| Response to reviewers on the paper titled “**Diagnostic delay of sarcoidosis: an integrated systematic review”** | | |
| --- | --- | --- |
| Reviewer 1 | | |
| Comments | Response | Manuscript edits |
| This appears to be a thorough and well-done systematic review of diagnostic delay in sarcoidosis. I appreciate the authors use of guidelines for systematic reviews and meta-analyses. Their work to combine and build conclusions from the various types of studies is very useful. I believe that this will be a helpful contribution to the literature and have made a few suggestions below that may improve the paper. | Thank you very much for your response. We greatly appreciate it. | |
| I appreciate the presentation of the MMAT quality screening in Supplementary table 2. In the results you indicate, "Consensus on the quality appraisal was low risk of bias; therefore, no study was excluded." It would be helpful to describe how the MMAT scores in Supp table 2 were used to form the "consensus" of low risk of bias. | Thank you for your comment. We agree that details on the consensus would be insightful for the readers. The edits have been made accordingly. | Page 9 line 214  After the double-quality appraisal, a consensus was reached by two authors regarding an overall low risk of bias for all studies; therefore, no study was excluded. |
| The methods state that, "Health systems were categorised as either gatekeeper (where primary care physicians authorise access to specialist physicians) or non-gatekeeper health systems, based on the health system in the country where the study was conducted." Could you clarify how this was determined for each country? I'm based in the United States where there are countless different private health insurers in addition to public insurers (with access limited), and some of these insurers use gatekeepers and some do not. It leaves me wondering where the US falls and how this was determined? | We appreciate your thorough comment. In countries which had a health system which normatively incorporates at a system level the use primary care gatekeepers, we classified them as “gatekeeper” countries. In the US, which has both open access and gatekeeper models, each paper was read by two authors to determine the model that applied to participants. Where this could not be determined the paper was not included (this was the case with the one US study by Judson 2003 which included participants from both systems)  As this might not have been explained well in the manuscript, we provided more details of this categorisation. | Page 7 Line 158  A country was classified as having a gatekeeper system if the systems of health financing uniformly used primary care gatekeepers, without the option of self-funding to see specialists, or models of health funding that supported open access to specialists. In countries with diverse health insurance models which may include open access and gatekeepers, such as the USA, an assessment was made for each publication by two authors. Where we could not determine the gatekeeper system used by participants the paper was excluded. |
| For the subgroup analyses presented in Supplementary Figures 2 through 4, it would be helpful to provide a bit of information on the statistical findings supporting the conclusions in the main text since many readers may not view the supplement. | We agree with your comment and are glad that you have mentioned this. We made the suggested edits to the methods and the results section. | |
| Lines 271 and 272 state, "Twenty-one studies reported treatment/diagnostic centres (2, 8, 25-27, 31-33, 35, 37-42, 44, 47-51)." I'm not sure what this means. Is this referring to sarcoidosis treatment/diagnosis centers first consulted? And how is this different from the multidisciplinary centers in the following statement? | Thank you for seeking clarification. It is the overall treatment or diagnostic centres. For this 19 referred to multidisciplinary centres and some research centres.  As this is confusing, we have made the sentence clearer. | Page 14 Line 325  Twenty-one studies reported visits to treatment or diagnostic centres including secondary or tertiary hospitals, research centres and university hospitals (2, 8, 25-27, 31-33, 35, 37-42, 44, 47-51). |
| I appreciate the figures presenting meta-aggregation results in Figures 3 and 4 as well as supplementary Figure 5. From the supplementary tables with this data, it looks like several of the factors/outcomes/symptoms were reported in more than one paper. Could the # of studies (or patients) where each was included be added to these figures to provide some information about their frequency in the literature? I see the articles for each factor/outcome/symptom are referenced in the text, but I think it would be helpful to represent numerically in the figures. | Thank you for the comment. The numbers have been added to Figure 3 and 4 as well as Supplementary Figure 5. | |
| It seems like there is some overlap in factors related to diagnostic delay and outcomes related to diagnostic delay. For example, both mention misdiagnosis and symptom progression. Indeed, I can see these fitting into both. Could you describe how you determined which section to include these findings for each study? Was it based on how it was framed in the original publication of the study? | Thank you for this comment. Yes, there are some overlaps, and we determined them based on how it was framed in the original publication of the study and through peer discussion amongst the authors. As this part is confusing, we have included details of it in the methods section. | Page9 Line 214  The broader categorisation of the aggregated data was decided through peer discussion and referral back to the original paper when needed. |
| It's an important finding that the authors did not identify any studies the examined patient or caregiver experiences of diagnostic delay. It appears that the overwhelming majority of papers included in the review were clinician reported, since they are case reports. It does look like at least one of the survey studies used patient-reported data. In the description of the studies, I would also be interested to see a description of the number of included studies that incorporate patient-reported data. | We agree that the case studies were the majority of the included studies. It would be interesting to see the use of patient-reported data. This information was extracted and added to Table 1 along with a part in the description of the included studies. | Page 10 Line 238  Twenty-eight of the included studies used non-patient-reported data including clinical reports and retrospective patient registry, while one used patient-reported data (45). |
| There is a fair amount of literature about diagnostic delay in many rare diseases and rare conditions in general. The authors cite studies on myositis and multiple sclerosis, but integration of a bit more of this literature might inform the discussion the potential impact of the delay and guide recommendations for future work on patient experiences of diagnostic delay in sarcoidosis. | Thank you for your comments. We have added broader integration of the literature around diagnostic delay in rare conditions in general in the discussion section. | Page 22 Line 511  Diagnostic delay can create a sense of uncertainty and, in many cases, escalating symptoms, as research examining people’s experiences with multiple sclerosis has found (55), placing them in a stressful state of ‘not knowing”. In children, delayed diagnosis has consequences for both children and their families, including anxiety, frustration and stress, fear of future reproduction due to ill-defined genetic risk (57). In rare diseases, hospitalisations and surgical interventions related to rare diseases are more frequent among people who experience a delayed diagnosis (58). Research examining experiences of hereditary angioedema found that inappropriate treatments were ineffective and at times, exacerbated the underlying condition (59). For some patients, symptoms were attributed to psychological reasons; due to this, some stopped seeking medical care despite severe symptoms (59). Attribution of rare disease symptoms to psychological or psychiatric reasons, and treatment in line with this is not uncommon; (57-59) however the impact of a rare disease on individuals’ mental health have important implications for the treatment and care of people with these health conditions (59).  Delay in diagnosis of sarcoidosis can cause impaired physical function, pain, reduced capacity to work, and strain on personal relationships, leading to a reduction in quality of life and the ability to engage in pleasurable activities, which in turn can have negative emotional consequences that impact wellbeing (60). A survey of the treatment priorities of people with sarcoidosis found that they most valued quality of life and functionality and concluded that psychological support was key to their wellbeing (61). Unfortunately, being able to discuss issues and concerns with clinician(s) about sarcoidosis cannot be realised until a diagnosis is received. |
| Reviewer #2 | | |
| This study aims to review the current literature to determine overall diagnostic delay of sarcoidosis, factors associated with diagnostic delay, and the experiences of people with sarcoidosis of diagnostic delay. This study provides presents a pooled analysis of diagnostic delay in all types of sarcoidosis to describe factors that are related to and associated with diagnostic delay. It also highlights the lack of evidence about the patient experience of diagnostic delay in sarcoidosis. | Thank you for your comments. | |
| Methods seem to focus only on how delay was found rather than also on factors associated with delay. | Thank you for your comment. The primary aim of this systematic review was to detect and review diagnostic delay and its associated factors. As the diagnostic delay pooling utilised a meta-analysis a heavy focus on statistical methods was given to assist the reader. No clear associations between diagnostic delay and independent variables were found in the literature. We identified factors that have been found to be related to diagnostic delay, although no clear associations. We have described these factors. | |
| Please provide reference for 'inverse variance weighted random effects model' | Thank you for pointing this out - we have added a reference for this. | Page 8 Line 189  We used an inverse variance weighted random effects model (Der-Simonian-Laird method) to pool mean diagnostic delay (6). |
| Under the Methods section, the data analysis section does not flow well and can be confusing. A restructure would be beneficial to make it clearer. This could benefit from presenting analysis by each aim - delay in diagnosis and factors associated with diagnosis separately under headings. Maybe also say here that there is no published evidence on experience here too. | Thank you for this comment. We have gladly made the suggested changes.  The methods section was restructured into 1) general data preparation, 2) analysis of diagnostic delay in sarcoidosis, and 3) analysis of symptoms, factors, outcomes and experiences in diagnostic delay in sarcoidosis.  A statement of not finding experiences was added as shown in the next column. | Page 10 Line 218  To our knowledge, none of the included studies reported data on experiences of diagnostic delay in sarcoidosis. |
